# Sequential RNA Polymerase II Activation Drives Human Hematopoiesis

**DOI:** 10.1016/j.celrep.2025.116802

**Published:** 2026-01-09

**Authors:** Derek H. Janssens, Christine A. Codomo, Dominik J. Otto, Lev Silberstein, Kami Ahmad, Steve Henikoff

**Affiliations:** 1 Department of Epigenetics, Van Andel Institute, Grand Rapids, MI, USA; 2 Basic Sciences Division, Fred Hutchinson Cancer Center, Seattle, WA, USA; 3 Howard Hughes Medical Institute, Chevy Chase, MD, USA; 4 Computational Biology Program, Fred Hutchinson Cancer Center, Seattle, WA, USA; 5 Translational Data Science IRC, Fred Hutchinson Cancer Center, Seattle, WA, USA; 6 Translational Science and Therapeutics Division, Fred Hutchinson Cancer Center, Seattle, WA, USA

## Abstract

Promoter-proximal pausing of RNA Polymerase II (Pol II) primes genes for rapid activation, yet how Pol II dynamics are temporally organized in adult stem cells to enable fast and flexible responses to environmental cues remains unknown. To address this, we developed sciCUT&Tag2in1 for joint profiling of Pol II and histone modifications in single cells. By profiling over 200,000 CD34^+^ hematopoietic stem cells (HSCs) and progenitors, we identify a Pol II regulatory cascade that directs the response to G-CSF-induced inflammatory stress. HSCs are activated by elevated Pol II occupancy and reduced Polycomb repression of immune response genes. Lineage commitment proceeds through sequential modes of Pol II activation, beginning with rapid pause-and-release genes, followed by slower initiate-and-release of Polycomb-repressed targets. sciCUT&Tag2in1 defines the temporal logic of how adult stem cells use paused Pol II to enable flexible lineage decisions, providing a powerful tool for studying the intersection of development, inflammation, and disease.

## Introduction:

The gene expression programs controlling embryonic development are largely hard-wired, but adult stem cell populations must dynamically respond to environmental stimuli to modulate their lineage outputs. The hematopoietic system is continually exposed to immune challenges, and in response hematopoietic stem cells (HSCs) must rapidly adapt by producing appropriate myeloid and lymphoid lineages to replenish effector cells and resolve inflammatory stress. This adaptive capacity relies on transcriptional plasticity orchestrated by RNA Polymerase II (Pol II), but how this flexibility is integrated with the epigenetic regulatory programs that establish and maintain heritable patterns of gene expression during lineage commitment remains poorly understood.

Transcription by Pol II is controlled through multiple steps, including loading, initiation, pausing, elongation and termination. Depending on the gene, different steps in this cycle are rate limiting and subject to regulatory inputs^1,2^. Promoter-proximal pausing of Pol II has been proposed to prime genes for rapid activation, but why some developmental genes are paused while others require de novo Pol II recruitment is not understood. Prior to immune activation, Pol II is paused at promoter-proximal regions of immune response genes and the large subunit of Pol II is phosphorylated on Serine 5 of the heptapeptide repeats in the C-terminal domain (CTD)^3^. Upon stimulation, kinases such as CDK9 phosphorylate Serine 2 on the Pol II CTD, triggering the rapid transcription of immediate early genes (IEGs), including the AP1 transcription factors JUN and FOS^4–6^. While extensive work has characterized the molecular cascades leading to immune gene activation in terminally differentiated cells, how these transcriptional events are integrated with epigenetic programs in HSCs remains unclear.

The Polycomb group (PcG) and Trithorax group (TrxG) chromatin-modifying complexes are indispensable for vertebrate hematopoiesis and regulate both the self-renewal and lineage-commitment programs that control hematopoietic stem cells (HSCs)^7–9^. These complexes instruct developmental gene expression patterns in part by catalyzing chemical modifications to the tails of nucleosomal histone proteins^10–13^. Depending on the genomic context, TrxG proteins catalyze the methylation of histone H3 on the lysine four residue to the mono (H3K4me1), di (H3K4me2) or tri (H3K4me3) methylated state^14^, and H3K4me3 deposition on gene promoters is important for the release of paused Pol II^13^. In embryonic stem cells, Pol II is poised on the promoters of numerous inactive genes that are decorated with the repressive modification histone H3 lysine 27 tri-methylation (H3K27me3) catalyzed by the PcG proteins^15^. However, previous studies relied on bulk chromatin profiling to generate an average across many different cells. Single-cell approaches are essential to determine how PcG and TrxG complexes coordinate Pol II regulation across diverse transcriptional states, and how these interactions shape gene activation kinetics in developing tissues.

Recent studies suggest repeated rounds of immune stimulation can induce “inflammatory memory” in HSCs, that involves the stable activation of IEGs^16^. In individuals with myeloproliferative neoplasms (MPN), this regulatory mechanism is disrupted, resulting in the hyperactivation of the IEGs^17^. Mutations in the PcG proteins ASXL1 and EZH2 are known to cause MPN^18–21^, and during mouse development PcG proteins dampen the stimulus-dependent transcription of IEGs by marking the gene body with H3K27me3^22^, suggesting that in MDS ASXL1 and EZH2 mutations may lead to de-repression of IEGs. However, technical challenges associated with profiling the chromatin landscape of heterogeneous samples have limited our ability to study PcG and TrxG proteins in HSCs, and the role of PcG proteins during inflammatory memory remains untested.

Emerging single-cell profiling technologies such as CUT&RUN and CUT&Tag have enabled mapping of chromatin-regulatory factors in heterogenous tissues on a genome-wide scale^23–27^. Single-cell multimodal strategies have typically focused on combining chromatin profiles with surface marker expression or RNA-seq^28,29^, but the direct integration of transcriptional machinery and chromatin regulatory proteins at single-cell resolution has not been achieved at scale.

Here, we develop sciCUT&Tag2in1, a single-cell combinatorial indexing (sci) method that leverages iterative rounds of cellular barcoding to simultaneously profile Pol II occupancy and histone modifications in up to 50,000 cells per experiment. We applied this method to profile over 200,000 human CD34+ hematopoietic stem and progenitor cells (HSPCs). Using G-CSF treatment during mobilization as an example of inflammatory challenge, we describe the coordinated regulation of Pol II by PcG and TrxG proteins during HSC differentiation. We find elevated Pol II occupancy over the IEGs directs HSC activation during inflammation. In addition, we demonstrate developmental pausing of Pol II in HSCs primes lineage-commitment genes for rapid activation during differentiation. This is followed by a slower Pol II initiate-and-release mechanism that activates Polycomb-repressed target genes. Our sciCUT&Tag2in1 method directly links transcriptional control and epigenetic regulation during the inflammatory response of human HSCs. This method will be invaluable for future studies of development, immune adaptation and disease in primary human tissues.

## Results:

### sciCUT&Tag2in1 enables simultaneous profiling of RNA Pol II and histone modifications in single cells.

**Figure 1.**
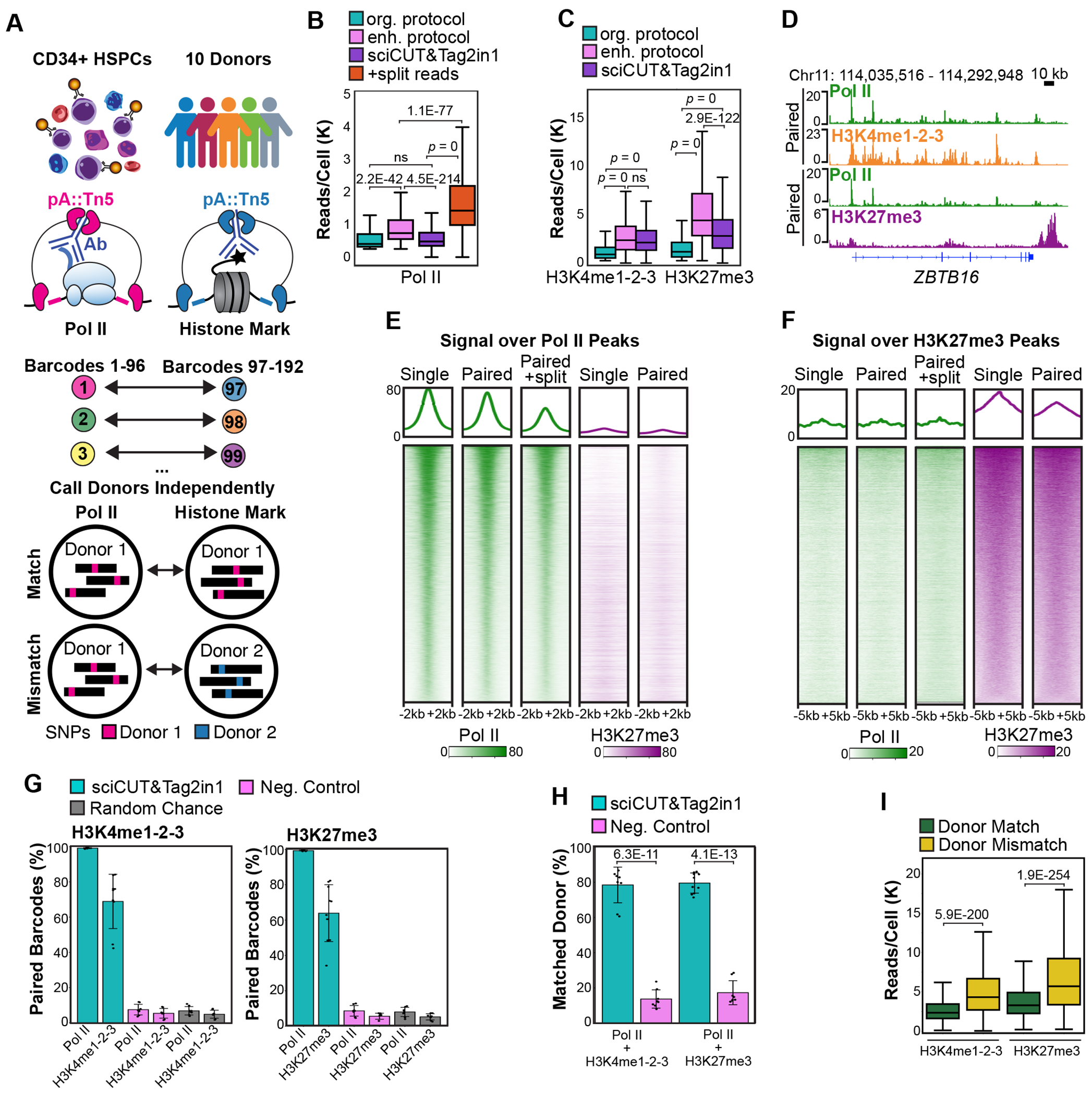
sciCUT&Tag2in1 pairs profiles of Pol II with histone modifications in single cells. (A) Schematic of the sciCUT&Tag2in1 workflow. CD34+ HSPCs mobilized from 10 healthy donors were labeled with pA-Tn5 transposase complexes targeting Pol II (Ser2P, Ser5P) in the first reaction and histone modifications (H3K4me1-2-3, H3K27me3) in the second reaction. Pol II is labeled with a first set of 96 barcodes that have a one-to-one correspondence with the barcodes (97-192) used to label histone marks, enabling independent assignment of reads corresponding to Pol II and histone marks from the same cell. Barcode pairing fidelity was assessed using SNP-based genotyping run independently on the two datasets. (B) Boxplots showing the number of reads per cell for Pol II profiling with the original protocol (*n* = 2,940 cells), enhanced protocol (*n* = 4,439 cells), and sciCUT&Tag2in1 protocol with and without split-barcode reads included (*n* = 70,546 cells). For boxplots here and throughout: center lines = median, box limits = first and third quartiles, whiskers = 1.5 times the interquartile range (IQR), *p*-values determined by an unpaired students t-test unless otherwise indicated. (C) Boxplots showing the number of reads per cell for H3K4me1-2-3 and H3K27me3 in the original, enhanced and sciCUT&Tag2in1 protocols. For H3K4me1-2-3 *n* = 41,273; 19,329 and 45,335 cells, respectively, and for H3K27me3 *n* = 37,295; 2,900 and 68,476 cells, respectively. (D) Genome Browser tracks over the *ZBTB16* locus show the distinct patterns of enrichment for Pol II, H3K4me1-2-3 and H3K27me3. Pol II profiles paired with H3K4me1-2-3 and H3K27me3 are very similar to one another. (E, F) Tornado plots showing the genomic-signal over 4-kb regions centered on Pol II peaks (E), or 10-kb regions centered and H3K27me3 peaks (F) for Pol II (green) and H3K27me3 (purple) datasets and demonstrating comparable signal quality for cells profiled individually (single) and paired by sciCUT&Tag2in1. (G) Bar plots showing the percentage of successfully paired barcodes for Pol II and histone marks. As a negative control, we attempted to identify matching barcodes from two independent biological replicates (pink) and compared this to the probability of finding matching barcodes based on random chance (grey). Whiskers = standard deviation of the mean; *n* = 8 biological replicates. (H) Bar graph of the fraction of matching Souporcell donor calls between the paired Pol II and histone mark data sets. The negative control is the matching barcodes from independent experiments (pink). Whiskers = standard deviation of the mean; *n* = 8 biological replicates. (I) Boxplots showing the reads per cell are significantly higher in the cells with mismatched donor calls, indicating these cells are likely doublets that were not called in one dataset. H3K4me1-2-3 barcodes matched *n* = 27,026 mismatched *n* = 5,781; H3K27me3 barcodes matched *n* = 32,490 mismatched *n* = 7,674.

Existing single-cell profiling methods are limited in their ability to capture Pol II regulatory dynamics together with the epigenetic programs concurrently within individual cells. To overcome this challenge, we developed sciCUT&Tag2in1, a single-cell combinatorial indexing approach that pairs genome-wide profiles of Pol II with histone modifications in single cells. We applied sciCUT&Tag2in1 to G-CSF-mobilized peripheral blood CD34+ HSPCs from ten healthy donors (Fig. 1A). To capture both the paused and elongating forms of RNA Pol II, we used a combination of antibodies targeting Serine-5 and Serine-2 phosphorylation on the Pol II CTD and performed tagmentation under low salt “CUTAC” (Cleavage Under Targeted Accessible Chromatin) conditions^30,31^. Histone marks were profiled during a second round of tagmentation and the protein-A-Tn5 (pA-Tn5) transposome was loaded with barcoded adapters to match Pol II profiles (barcodes 1-96) with histone marks (barcodes 97-192; Fig. 1A, Supplementary Fig. 1A, Supplementary Table 1). To capture developmental repression by PcG proteins, we profiled H3K27me3. Genomic features decorated with H3K4 methylation are often found in close proximity to one another and coordinate the regulation of individual genes^14^. To capture the full “TrxG-protein regulome” in a single reaction, we used a combination of antibodies targeting all three H3K4-methylation states (H3K4me1-2-3). This workflow enables scalable profiling of Pol II together with chromatin landscapes, establishing a platform to investigate coordinated gene regulation at single-cell resolution.

To optimize data recovery from whole cells, we loaded pA-Tn5 with PAGE-purified DNA adapters for tagmentation and included thermolabile proteinase K in the chromatin release buffer. These improvements more than doubled the number of unique reads per cell for Pol II, H3K4me1-2-3 and H3K27me3 and enhanced the signal-to-noise ratio, as measured by the fraction of reads in peaks (FRiPs) (Fig. 1B,C, Supplementary Fig. 1B–D). When profiled in combination with one another, the sciCUT&Tag2in1 method yielded a median of 472 Pol II, 2133 H3K4me1-2-3 and 2761 H3K27me3 reads per cell (Fig. 1B,C). To prevent cross-reaction between the primary antibodies used to label histone proteins in the second round of tagmentation and the unbound Fab domains of the secondary antibodies used to label Pol II during the first round of tagmentation, sciCUT&Tag2in1 includes a blocking step with the Fc portion of the rabbit IgG (Supplementary Fig. 1A). This blocking step is sufficient to minimize cross-contamination due to integration of two opposing adapters from the second round of tagmentation at sites bound by Pol II (see below). However, the sciCUT&Tag2in1 method still produces a substantial number of “split-barcode reads” that include one adapter inserted near Pol II during the first round of tagmentation and a paired adapter inserted during the second round of tagmentation. Because the first adapter must have been inserted cleanly at Pol II bound sites, to err on the side of caution, rather than interpreting these split-barcodes reads as sites of true co-occupancy, we combined these reads with the Pol II datasets which resulted in a significant boost in the median number of reads per cell (1397; Fig. 1B). As expected, the Pol II, H3K4me1-2-3 and H3K27me3 profiles showed differential patterns of enrichment across the genome, and Pol II profiles paired with either H3K4me1-2-3 or H3K27me3 were very similar to one another (Fig. 1D). Importantly, we observed minimal cross-contamination in the sciCUT&Tag2in1 profiles, with little H3K27me3 signal over the Pol II signal peaks, and vice versa, and this was comparable to the amount of overlap when Pol II and H3K27me3 were profiled individually (Fig. 1E,F). We conclude that sciCUT&Tag2in1 maintains the exceptional quality and specificity of the CUT&Tag method.

To validate barcode pairing fidelity, we profiled a mixture of 10 donors, and assessed the frequency of matching SNP-based donor assignments by Souporcell^32^ run independently on the Pol II and histone mark datasets (Fig 1A). Based on a series of control reactions that included a limited number of donors, we found the Souporcell single-cell genotyping tool was able to accurately identify cells from eight of the ten donors we profiled (Supplementary Fig. 1E). For nearly all Pol II-profiled cells, the matching barcode was also detected in the paired H3K4me1-2-3 and H3K27me3 profiles and for 60-70% of H3K4me1-2-3 and H3K27me3 cells the matching barcode was present in the Pol II profiles (Fig. 1G). As a negative control, we assessed barcode overlap between Pol II and histone modifications profiled in separate experiments and found that the fraction of matching barcodes corresponded to the level expected by random chance (Fig. 1G). We then examined donor assignment concordance and found that Souporcell assigned the same donor to ~80% of the Pol II and histone mark barcode pairs, and this was significantly higher than the 15-20% concordance observed in the negative control (Fig. 1H). Donor mismatches occurred when cells were called as a single donor based on one dataset but were called as a doublet in the other dataset (Supplementary Fig. 1F), suggesting insufficient SNP coverage in one of the profiles prevented Souporcell from detecting the doublet. Consistent with this interpretation, cells with mismatched donor calls also had higher reads per cell than those with matching calls (Fig. 1I). Together, these results demonstrate sciCUT&Tag2in1 enables robust, scalable, and accurate joint profiling of Pol II and histone marks at single-cell resolution, providing a powerful tool to study gene regulation in heterogenous mixtures of CD34+ HSPCs.

### sciCUT&Tag2in1 links lineage identity to immune activation and cell cycle states

**Figure 2.**
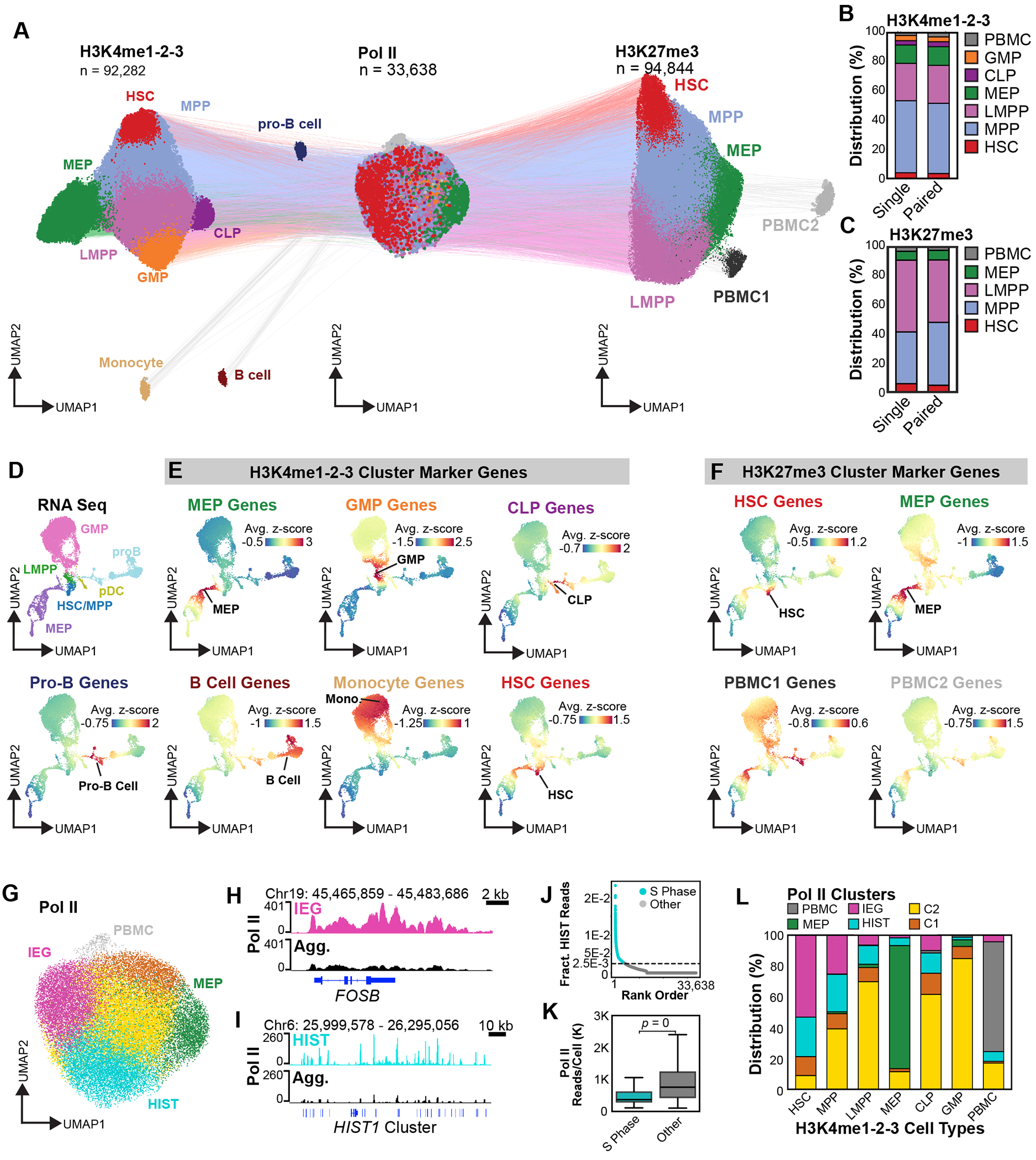
sciCUT&Tag2in1 links lineage identity to immune activation and cell cycle states. (A) Joint UMAP embedding of H3K4me1-2-3, Pol II, and H3K27me3 profiles. Connecting lines indicate joint profiles from the same cell. The H3K4me1-2-3 and H3K27me3 UMAPs are colored by the inferred cell types (see below), and the Pol II UMAP is colored according to the paired H3K4me1-2-3 and H3K27me3 cell-type annotations. (B,C) Barplots showing the distribution of major hematopoietic stem and progenitor cell types in single versus paired H3K4me1-2-3 profiles (B), and H3K27me3 profiles (C). (D) A previously generated UMAP embedding of lineage-depleted bone marrow profiled by RNA-seq. (E,F) RNA-seq UMAP from (D), colored by the average z-score distributions of cluster-specific marker genes from the H3K4me1-2-3 profiles (E) and H3K27me3 profiles (F). (G) UMAP embedding of Pol II single-cell profiles colored by Leiden clusters. (H) Genome browser track showing Pol II enrichment over immediate early gene loci (*FOSB*) within the IEG cluster. (I) Genome browser track showing Pol II enrichment over the *HIST1* cluster. (J) Rank order plot showing Pol II enrichment over the HIST cluster genes; cells with a fraction of reads above the threshold (dotted line) were called as S-phase cells (blue). (K) Boxplot comparing total Pol II reads per cell between S-phase and non-S-phase cells (*n* = 4,227 and 101,978 cells, respectively), demonstrating a global decrease in Pol II loading occurs during S-phase. *p*-value is from a two-sided Mann-Whitney U test. (L) Barplots showing distribution of Pol II clusters across cell types defined by H3K4me1-2-3 annotations.

Having established the scalability and precision of sciCUT&Tag2in1 for joint profiling, we next sought to map the differentiation state of human HSPCs based on the Pol II status and chromatin landscape. To enrich for high quality profiles of single cells, we performed a series of quality control (QC) steps on the H3K4me1-2-3, H3K27me3 and Pol II datasets that included batch correction using Harmony^33^, followed by iterative rounds of high-resolution clustering and SNP-based doublet removal (Supplementary Fig. 2 A–E). In humans, Polycomb group proteins play a critical role in dosage compensation^34^, and for UMAP embeddings of H3K27me3, we excluded reads from the X and Y chromosomes to avoid donor separation based on the fraction of X chromosome reads (Supplementary Fig. 2A). H3K4me1-2-3 profiles initially formed clusters that were driven by signal over a small number of repetitive features, and we identified 95 genomic regions that were added to the ArchR^35^ blacklist and were not considered during subsequent embeddings (Supplementary Fig. 2B–D). After QC, we recovered 92,282 high-quality H3K4me1-2-3 single-cell profiles, 94,844 H3K27me3 single-cell profiles, and 33,638 single-cell Pol II profiles, of which 6,677 were paired with H3K4me1-2-3, and 13,543 were paired with H3K27me3 (Fig. 2A). Importantly, sciCUT&Tag2in1 profiles co-embedded evenly in UMAP space alongside cells profiled for H3K4me1-2-3 or H3K27me3 individually (Fig. 2B,C, Supplementary Fig. 2F,G).

To annotate our single-cell H3K4me1-2-3 profiles (Fig. 2A), we began with low-resolution clustering and cross-referenced the ArchR^35^ imputed marker genes with a single-cell RNA-seq dataset collected from lineage-depleted bone marrow^36^ (Fig. 2D). The ArchR suite calculates gene scores for individual cells according to the weighted values of reads that fall in the gene transcriptional start site (TSS), gene body and the surrounding distal regulatory elements^35^. Gene scores are then imputed in neighboring cells^37^, and marker genes are identified based on significant increases or decreases in each of the assigned clusters relative to the aggregate data. Because the H3K4me1-2-3 marks are associated with transcriptional activation, we focused our analysis on marker genes that had cluster-specific increases in the H3K4me1-2-3 gene scores. This strategy identified a rich collection of marker genes associated with hematopoietic lineage commitment (Supplementary Table 2). Marker genes from the major H3K4me1-2-3 clusters where highly expressed in the megakaryocyte erythroid progenitors (MEPs), granulocyte monocyte progenitors (GMPs), and common lymphoid progenitors (CLPs), consistent with their classification as early lineage-committed progenitors (Fig. 2E). Marker genes from satellite clusters in the H3K4me1-2-3 UMAP were expressed in pro-B cells, differentiated B cells and monocytes (Fig. 2D,E). In the red H3K4me1-2-3 cluster, we identified 44 marker genes, and while these did not include classic markers of HSCs, the 21 genes that are expressed above the minimal detection threshold are enriched in HSCs as annotated by RNA-seq (Fig. 2E, Supplementary Table 2). These findings demonstrate that single-cell H3K4me1-2-3 profiles resolve early hematopoietic progenitors and their progression along distinct lineage trajectories.

Next, we annotated the single-cell H3K27me3 UMAP embedding. Because H3K27me3 is associated with developmental gene repression, we focused on ArchR-imputed marker genes with significant cluster-specific reductions in H3K27me3 scores, indicative of potential gene activation. In the red cluster, H3K27me3 was significantly depleted at *PRDM16* and *MECOM* (Supplementary Table 2), two transcription factors essential for HSC self-renewal^38–41^. Of the top 75 most H3K27me3-depleted marker genes in this red cluster, 47 were expressed above the RNA-seq detection threshold and enriched in the HSC cluster at the root of the RNA-seq UMAP (Fig. 2F). Our H3K27me3 analysis also identified marker genes enriched in the MEP branch of the RNA-seq UMAP, but not the GMP and lymphoid lineages (Fig. 2F). H3K27me3-low marker genes from the two satellite clusters in the H3K27me3 UMAP were distributed across all three primary hematopoietic lineage trajectories in the RNA-seq UMAP (Fig. 2F), leading us to annotate them as peripheral blood mononuclear cells 1 and 2 (PBMC1 and PBMC2). Together these results support a recently proposed model^42^, in which repressive histone modifications such as H3K27me3 reflect a cell’s position along the HSC-differentiation axis, while activating marks such as H3K4me1-2-3 provide finer resolution of lineage-specific trajectories in differentiating progenitors (Supplementary Fig. 2H). In addition, the overall concordance of the developmentally regulated chromatin landscapes we observe and the lineage-specific gene expression programs from single-cell RNA-seq of whole bone marrow suggests that key aspects of hematopoietic differentiation are preserved in the G-CSF mobilized CD34+ fraction we profiled.

The overall structure of the Pol II UMAP embedding was relatively simple, and coloring cells by their paired H3K4me1-2-3 and H3K27me3 annotations revealed clear separation of MEPs and PBMCs into distinct clusters (Fig. 2A). We partitioned the Pol II UMAP into six clusters and identified marker genes using ArchR-imputed gene scores (Fig. 2G, Supplementary Table 2). One cluster showed strong Pol II enrichment over the Immediate Early Genes (IEGs), including the AP-1 transcription factors *FOSB*, *FOS* and *JUN* as well as *DUSP1, KLF2, NFATC1*, and *EGR1* (IEG cluster; Fig. 2G,H, Supplementary Table 2). Another cluster was defined by Pol II occupancy at replication-coupled histone genes located in the large *HIST1* locus on chromosome 6, with additional but lower enrichment at the *HIST2* locus on chromosome 1 (HIST cluster; Fig. 2G,I, Supplementary Fig. 2I, Supplementary Table 2). These patterns indicate that Pol II occupancy reflects both cell identity and dynamic transcriptional states, including cytokine-induced activation and cell cycle progression.

A recent study developed single-cell Pol II run-on sequencing to detect nascent RNAs and demonstrated that histone gene transcription is synchronized during S Phase^43^. To identify S-phase cells in our sciCUT&Tag2in1 dataset, we calculated the fraction of total Pol II reads that mapped to histone genes and classified cells with high values in the HIST cluster as S-phase (Fig. 2J). Interestingly, these cells showed a significant reduction in total Pol II reads per cells (Fig. 2K). These results build on the proposed model that global transcription is downregulated during DNA replication to reduce replication-transcription conflicts and DNA damage^43^, and indicate that S-phase transcription is reduced by limiting the first step of the transcription cycle, the loading of Pol II at promoters.

Analysis of cell type distributions across Pol II-defined clusters revealed strong enrichment of HSCs in the IEG cluster, and higher frequencies of HSCs and MPPs in the HIST cluster relative to other hematopoietic populations (Fig. 2L, Supplementary Fig. 2J). In the bone marrow, HSCs typically reside in a quiescent state. In response to G-CSF mobilization, we find HSCs are in an activated state, and Pol II is enriched over markers of an acute immune response that likely promotes cellular division, as indicated by an increased S-Phase index. We conclude joint profiling with sciCUT&Tag2in1 enables integrated analysis of cell fate and cell state through simultaneous measurement of transcriptional activity and chromatin landscapes.

### Immediate Early Genes are highly transcribed in HSCs and subject to Polycomb-mediated repression during differentiation.

**Figure 3.**
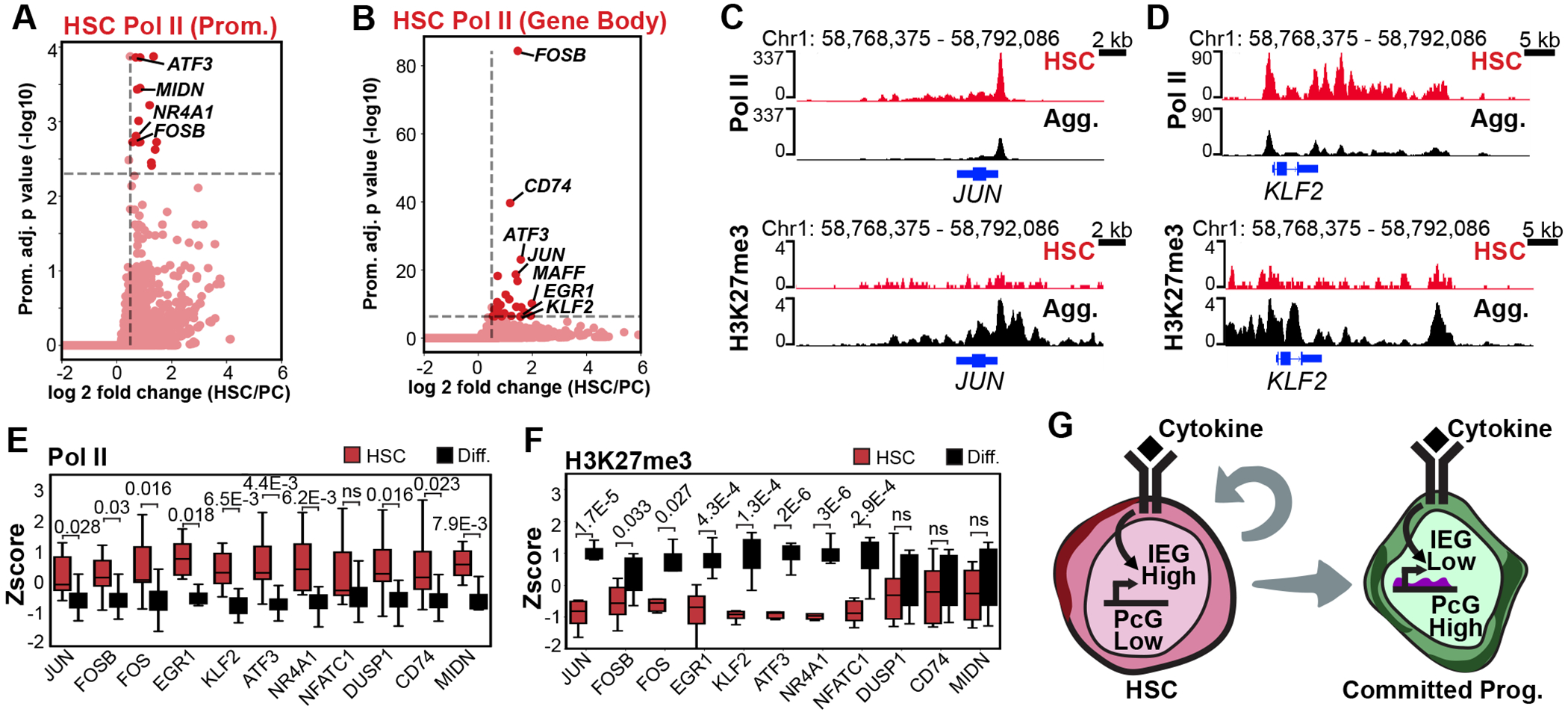
Coordinated regulation of immediate early genes by Pol II and Polycomb in HSCs. (A) Volcano plot showing the enrichment of Pol II over Promoters for all genes in the H3K27me3 annotated HSC cluster. (B) Same as (A) but showing the enrichment of Pol II over Gene Bodies. (C) Genome browser tracks showing the Pol II signal (top) and H3K27me3 signal (bottom) over the *JUN* locus. (D) Same as (C) but showing the signals over the *KLF2* locus. (E) Boxplots showing the relative enrichment of Pol II over the IEGs in HSCs (red) versus the other progenitor cells we profiled (Diff., black boxes); *n* = 7 pseudobulk replicates paired with H3K4me1-2-3 or H3K27me3; *p*-values from a one-sided students t-test. (F) Same as (E) but showing the relative depletion of H3K27me3 over the IEGs in HSCs (red); *n* = 6 pseudobulk replicates; *p*-values from a one-sided students t-test. (G) In response to inflammatory stress, IEGs are prone to enhanced Pol II transcription in HSCs, where they lack Polycomb-mediated repression (PcG).

Given the enrichment of HSCs in the IEG cluster and prior evidence that cytokine-responsive HSCs sustain IEG activation as part of an inflammatory memory response^16^, we asked whether G-CSF mobilization triggers a similar transcriptional program. For each gene, we defined the promoter as the annotated TSS with the highest Pol II enrichment in aggregate data and designated the region from this TSS to the transcription termination site as the gene body. In both the H3K4me1-2-3 and H3K27me3 sciCUT&Tag2in1 datasets IEGs ranked among the genes with the highest HSC-specific Pol II enrichment across both promoters and gene bodies, including many of the IEGs we previously identified as marker genes in the Pol II cluster, as well as the IEGs *NR4A1* and *ATF3* (Fig. 3A,B, Supplementary Fig. 3A,B). Interestingly, Pol II was also strongly enriched at *MIDN*, which encodes Midnolin—a protein that promotes proteasomal degradation of IEG proteins through a non-canonical, ubiquitination-independent pathway^44,45^. This suggests a feedback mechanism in HSCs that couples IEG transcriptional activation with rapid protein turnover for tight control of proliferative responses to immune stimulation.

During mouse development, PcG proteins mark the gene body of numerous IEGs with H3K27me3 to dampen their stimulation-dependent transcription^22^, and so we next asked whether PcG proteins may also play a role in the regulation of IEGs in human HSCs. By inspecting the genome browser tracks, we found that in addition to accumulation of Pol II, HSCs displayed reduced H3K27me3 levels over many of the IEGs (Fig. 3C,D). Quantitative comparisons of the set of HSC-enriched IEGs we identified confirmed a significant increase in Pol II occupancy over these IEGs as well as *MIDN* in HSCs accompanied by a decrease in H3K27me3 over these IEGs with the exception of *DUSP1* and *CD74*, which show low levels of H3K27me3 in committed progenitors cells as well (Fig. 3E,F). We did not observe any changes in the H3K4me1-2-3 levels over IEGs between HSCs and committed progenitors (Supplementary Fig. 3C). These results suggest that as compared to committed progenitors, HSCs may be particularly sensitive to inflammatory stress because Polycomb-mediated repression of the IEGs is reduced (Fig. 3G).

To examine how this Polycomb-mediated repression of IEGs may be related to inflammatory memory in HSCs, we examined Pol II and H3K27me3 enrichment over the rest of the transcription factors that are most highly induced in response to repeated rounds of dual stimulation with the cytokine TNFa and bacterial lipopolysaccharide^16^. This analysis revealed Pol II is also significantly elevated over the IEGs *MAFF* and *FOSL1* in CD34+ mobilized HSCs, however we observed no significant change in H3K27me3 (Supplementary Fig. 3D,E). Together, five of the thirteen IEGs that showed elevated Pol II in G-CSF mobilized HSCs are also strongly activated in HSCs in the repeated dual immune stimulation model (Supplementary Fig. 3F). Although our study cannot assess persistence directly, the observed reduction in H3K27me3 at key IEG loci suggests that G-CSF mobilization may initiate epigenetic remodeling events that could influence future HSC responses to immune stimuli. We conclude that distinct inflammatory signals likely activate partially overlapping but distinct sets of IEGs in HSCs.

### Self-renewal genes retain promoter-proximal Pol II during stem cell differentiation.

**Figure 4.**
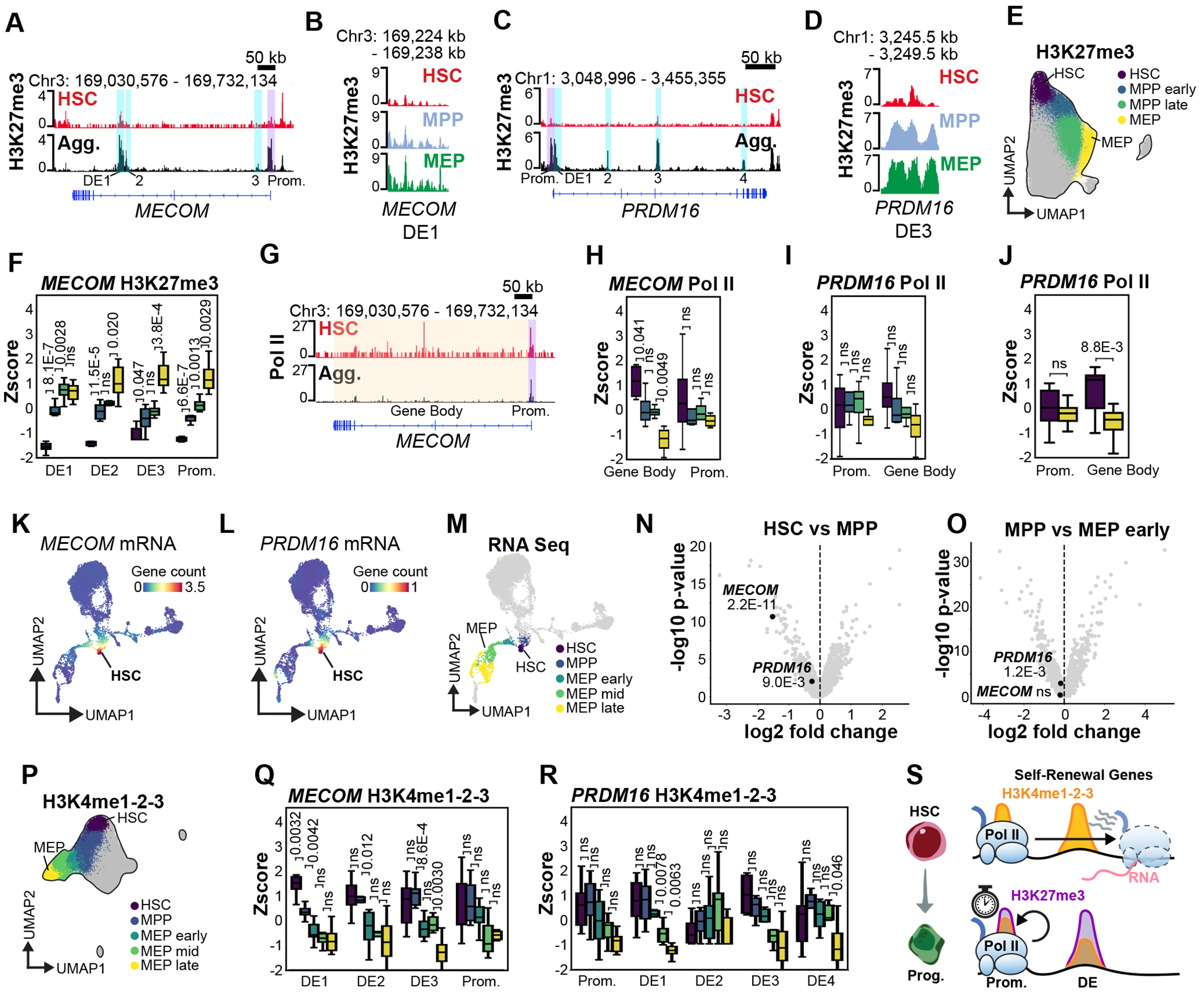
Self-renewal genes gain H3K27me3 during stem cell differentiation but retain promoter-proximal Pol II. (A) Genome browser track showing the H3K27me3 signal over the *MECOM* locus. The distal elements (DE1-3, blue) and Promoter (purple) are highlighted. (B) Expanded track showing H3K27me3 signals over *MECOM* DE1 in the HSC, MPP and MEP clusters. (C) Same as (A) showing the *PRDM16* locus. (D) same as (B) showing *PRDM16* DE3. (E) H3K27me3 UMAP showing clusters along the HSC-to-MEP trajectory. (F) Boxplot showing the relative enrichment of H3K27me3 over the *MECOM* regulatory elements across the HSC-to-MEP trajectory, *n* = 6 pseudobulk replicates. (G) Genome browser tracks showing the Pol II signal over the *MECOM* locus. (H,I) Same as (F) showing the Pol II signal over the *MECOM* Promoter and Gene Body (H) and the *PRDM16* Promoter and Gene Body (I); *n* = 4 pseudobulk replicates paired with H3K27me3; *p*-values from a one-sided students t-test. (J) Same as (I) but *n* = 7 pseudobulk Pol II replicates paired with H3K27me3 and H3K4me1-2-3 and comparisons are made between HSCs and MEPs directly. (K-M) RNA-seq UMAP colored by the imputed number of mRNA reads for *MECOM* (K) and *PRDM16* (L), and cells split into clusters along the HSC-to-MEP trajectory (M). (N) Differential gene expression analysis comparing the HSC and MPP clusters from (M). (O) Same as (N) comparing the MPP and MEP early clusters. (P) H3K4me1-2-3 UMAP showing clusters along the HSC-to-MEP trajectory. (Q,R) Same as (F) but showing the relative enrichment of H3K4me1-2-3 along the HSC-to-MEP trajectory assigned in (P) over the *MECOM* (Q) and *PRDM16* (R) regulatory elements; *n* = 6 pseudobulk replicates. (S) During HSC differentiation, H3K27me3 accumulates over the regulatory elements of self-renewal genes marked by H3K4me1-2-3; in the H3K27me3-marked repressed state, Pol II remains paused on the promoters.

Prompted by the coordinated gain of Pol II and loss of H3K27me3 at IEGs, we next examined whether similar changes in chromatin—including Pol II, H3K4me1-2-3, and H3K27me3—occur at genes regulating HSC self-renewal and lineage commitment. *MECOM* and *PRDM16* are essential for HSC self-renewal^38–41^, and we identified a collection of promoter and distal regulatory elements (DEs) at these loci that exhibited pronounced increases in H3K27me3 during HSC differentiation (Fig. 4A–D). To examine the relative timing of H3K27me3 accumulation, we partitioned cells into four clusters along the HSC-to-MEP trajectory (Fig. 4E). This revealed distinct kinetics of H3K27me3 gain across individual DEs. At the *MECOM* locus, DE1, which overlaps the internal short isoform promoter, gained H3K27me3 first, and this was completed prior to MEP commitment, while the long isoform promoter, DE2, and DE3 accumulated H3K27me3 more gradually throughout the MPP and MEP stages (Fig. 4B,F). Similarly, DEs at the *PRDM16* locus gained H3K27me3 at different rates, with DE3 showing the most rapid increase, completed by early MPP commitment (Fig. 4D, Supplementary Fig. 4A).

By examining the H3K27me3 paired data, we found Pol II was reduced across the *MECOM* gene bodies during HSC-to-MEP lineage commitment, however promoter-associated Pol II remained relatively stable, suggesting pause release is inhibited (Fig. 4G,H). We observed a similar maintenance of Pol II occupancy at the *PRDM16* promoter throughout the HSC-to-MEP trajectory, with a more gradual reduction in Pol II over the gene body that was only significant when the levels were compared between HSCs and MEPs and Pol II data paired with both H3K27me3 and H3K4me1-2-3 was included (Fig. 4G,H, Supplementary Fig. 4B). To determine how these alterations in the Pol II occupancy are related to gene expression, we examined single-cell RNA seq data and confirmed that *MECOM* and *PRDM16* transcripts are both downregulated during HSC commitment (Fig. 4K,L). Next, we performed differential gene-expression between clusters of cells along the HSC-to-MEP trajectory (Fig. 4M) and found that *MECOM* expression is more highly expressed in HSCs and more rapidly repressed than *PRDM16* during MEP differentiation (Fig. 4N,O, Supplementary Fig. 4C,D). We conclude that changes in Pol II occupancy across the gene bodies of *MECOM* and *PRDM16* are indicative of the transcriptional repression of these genes during HSC differentiation.

Given that H3K4me3 deposition contributes to Pol II pause release^13^, we next examined changes in H3K4me1-2-3 at the same regulatory elements (Supplementary Fig. 4E,F). Cells from the H3K4me1-2-3 UMAP were partitioned into five clusters along the HSC-to-MEP lineage trajectory (Fig. 4P). While H3K4me1-2-3 levels declined at *MECOM* DE1, DE2, and DE3, as well as *PRDM16* DE1, at distinct rates, H3K4me1-2-3 levels over the *MECOM* and *PRDM16* promoters remained stable throughout differentiation (Fig. 4Q,R). Although H3K4me1-2-3 signal was maintained at the *MECOM* and *PRDM16* promoters in MEPs, Pol II fails to transition into productive elongation and RNA expression is repressed. This suggests that H3K4 methylation alone is insufficient to drive pause release in the presence of newly acquired H3K27me3, consistent with Polycomb-mediated repression that blocks transcriptional activation of self-renewal genes (Fig. 4S).

### Pol II is activated through distinct mechanisms on MEP-commitment genes.

**Figure 5.**
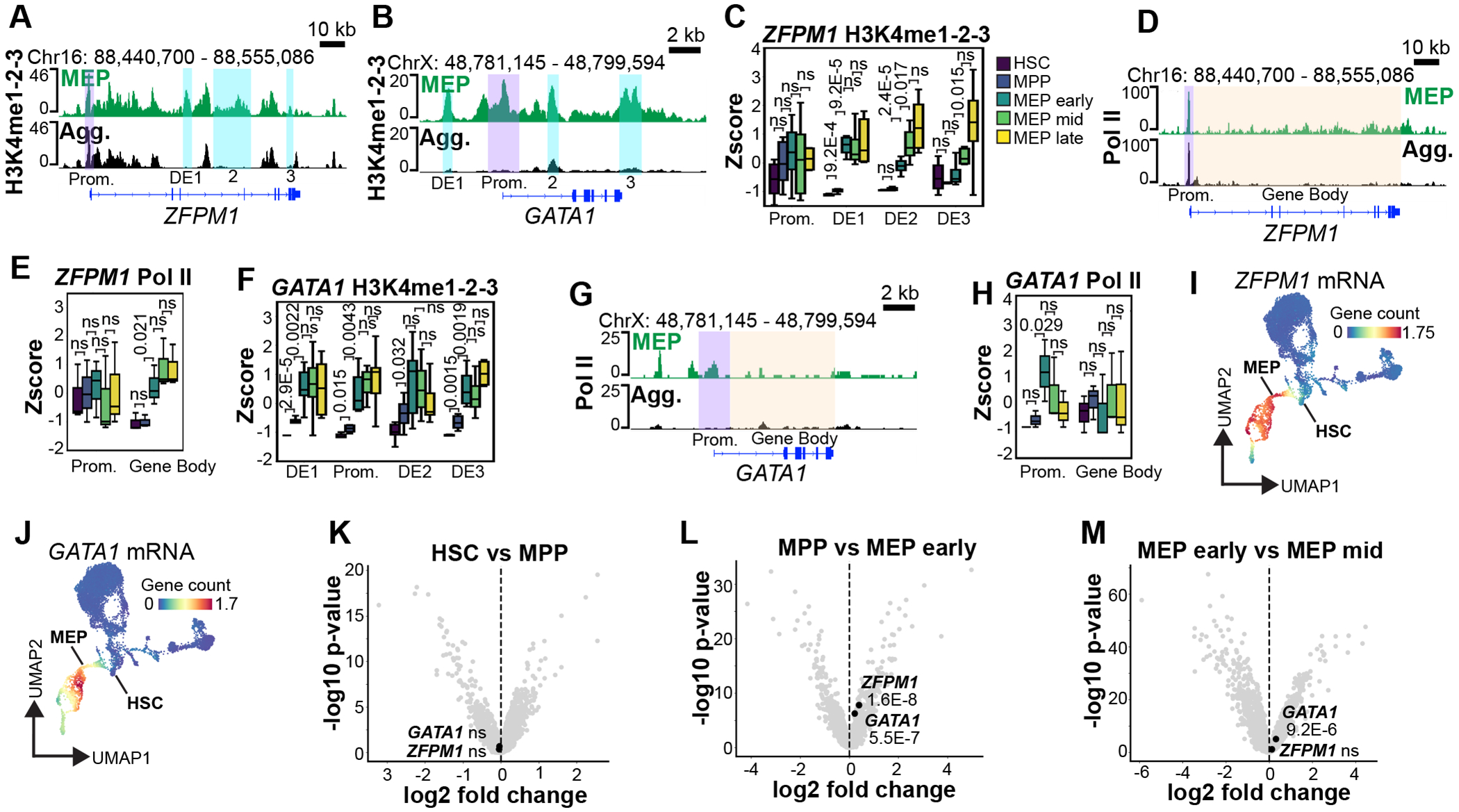
Pol II is activated through distinct mechanisms on MEP-commitment genes. (A) Genome browser track showing the H3K4me1-2-3 signal over the *ZFPM1* locus. The distal elements (DE1-3, blue) and Promoter (purple) are highlighted. (B) Same as (A) but for the *GATA1* locus. (C) Boxplot showing the relative enrichment of H3K4me1-2-3 over the *ZFPM1* regulatory elements across the HSC-to-MEP trajectory; *n* = 6 pseudobulk replicates. (D) Genome browser track showing the Pol II signal over the *ZFPM1* locus. (E) Same as (C) but showing the Pol II signal over the *ZFPM1* Promoter and Gene Body; *n* = 4 pseudobulk replicates paired with H3K4me1-2-3; *p*-values from a one-sided students t-test. (F) Same as (C) but comparing the H3K4me1-2-3 signals over the *GATA1* regulatory elements. (G) Genome browser track showing the Pol II signal over the *GATA1* locus. (H) Same as (C) but showing the Pol II signal over the *GATA1* Promoter and Gene Body. (I,J) RNA-seq UMAP colored by the imputed number of mRNA reads for *ZFPM1* (I) and *GATA1* (J). (K-M) Differential gene expression analysis comparing the HSC and MPP clusters (K), the MPP and MEP early clusters (L) and the MEPearly and MEP mid clusters (M).

To determine how Pol II and chromatin dynamics are coordinated during lineage-commitment, we initially focused on the HSC-to-MEP lineage trajectory. The transcription factors *ZFPM1* and *GATA1* function together to drive MEP lineage commitment^46–49^, and exhibited stepwise activation of the surrounding regulatory elements, marked by gains in H3K4me1-2-3 (Fig. 5A,B). At the *ZFPM1* locus, the promoter was already marked by H3K4me1-2-3 in HSCs and remained stable, whereas DE1 and DE2 gained H3K4me1-2-3 early during MEP commitment, followed by delayed activation of DE3 (Fig. 5C). In parallel, Pol II was enriched at the *ZFPM1* promoter but absent from the gene body in HSCs, consistent with promoter-proximal pausing (Fig. 5D–E), and during early MEP commitment, gene body Pol II occupancy increased significantly (Fig. 5D–E). H3K27me3 was undetectable at the *ZFPM1* locus throughout the trajectory (Supplementary Fig. 5A,B). Together, these data suggest Pol II is held in a paused confirmation on the *ZFPM1* promoter in the absence of Polycomb-mediated repression, and that release of paused Pol II into the gene body coincides with H3K4me1-2-3 accumulation at distal regulatory elements.

In contrast, *GATA1* underwent a distinct cascade of molecular events during transcriptional activation. H3K4me1-2-3 accumulated more rapidly at multiple DEs, beginning in the MPP stage, prior to overt lineage commitment (Fig. 5F). In HSCs, Pol II was absent at the *GATA1* locus but was recruited *de novo* to the promoter during early MEP commitment (Fig. 5G,H, Supplementary Fig. 4C). H3K27me3 was enriched over the *GATA1* locus and gradually declined over the promoter and DE2 during MEP commitment (Supplementary Fig. 5C,D). By comparing the RNA expression dynamics of *ZFPM1* and *GATA1*, we found these genes are detectable in very few HSCs and MPPs and are then upregulated rapidly during MEP commitment (Fig. 5I–M, Supplementary Fig. 5C,D). *ZFPM1* shows slightly stronger activation during early MEP commitment, and *GATA1* expression continued to increase during mid MEP commitment (Fig. 5L,M). These observations suggest the promoter proximal pausing of Pol II on *ZFPM1* contributes to rapid activation during lineage commitment and that *GATA1* is activated through a distinct mechanism, involving Pol II recruitment followed by rapid release.

### Sequential Pol II pause-and-release and initiation-and-release mechanisms drive lineage commitment.

**Figure 6.**
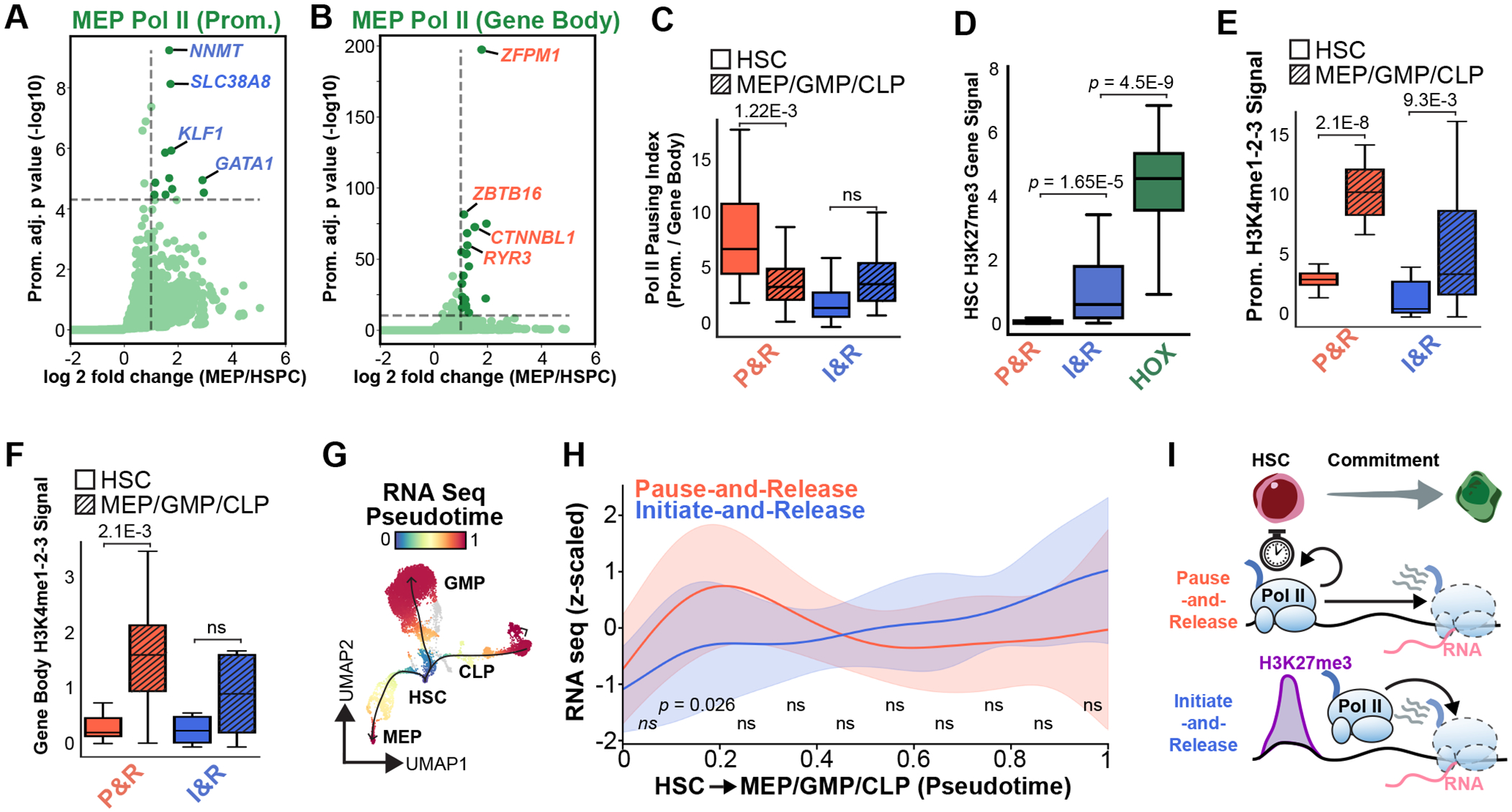
Sequential Pol II pause-and-release and initiation-and-release mechanisms drive lineage commitment. (A, B) Volcano plots showing the differential Pol II occupancy in MEPs relative to HSPCs over Promoters (A) and Gene Bodies (B). Lineage-specific genes with significant Pol II enrichment are highlighted in blue (promoter) or red (gene body). (C) Boxplots showing the Pol II pausing index (Promoter / Gene Body Pol II) across HSCs and lineage-committed MEPs, GMPs, and CLPs for pause-and-release (P&R; *n* = 25 genes) and initiate-and-release (I&R; *n* = 19 genes) genes. (D) Boxplots showing H3K27me3 levels over P&R, I&R, and HOX genes in HSCs. (E) Boxplots comparing promoter H3K4me1-2-3 signal in HSCs and lineage-committed cells for P&R and I&R genes. (F) Same as (E), showing H3K4me1-2-3 signal over gene bodies. (G) RNA-seq UMAP showing pseudotime trajectories for HSCs differentiating into MEP, GMP, and CLP lineages. (H) Line plot showing the average expression of P&R (red) and I&R (blue) genes across pseudotime trajectories normalized from 0 (HSC) to 1 (terminal lineage state). Expression values are calculated as the average z-scaled expression of each gene along its respective lineage. (R) During lineage commitment, Pol II transcription is rapidly activated through a pause-and-release mechanism, followed by a slower initiated-and-release activation of Polycomb-repressed genes.

We were intrigued by the two distinct modes of Pol II activation and asked whether these mechanisms extended to other genes across the MEP, GMP and CLP lineages. We began by identifying genes that preferentially gain Pol II signal over either the promoter or the gene body during differentiation. Genes were selected by differential analysis and thresholding, with a more permissive cutoff applied to GMPs due to their lower representation in the CD34+ compartment. This approach identified 25 genes that gain Pol II over the gene body (MEP = 21, GMP = 3, and CLP =1), and 19 genes with increase Pol II promoter occupancy (MEP = 11, GMP = 7 and CMP =1; Fig. 6A,B, Supplementary Fig. 6A–D). As expected from our previous observations, in the MEP lineage *ZFPM1* was captured in the group that gains Pol II over the gene body, and *GATA1* was included in the promoter group (Fig. 6A,B). Using this classification method, the promoter and gene body groups were largely non-overlapping, providing further support for two distinct modes of transcriptional activation during hematopoietic lineage-commitment, and the only shared gene– *ANK1*–was removed from downstream analysis.

To quantitatively assess promoter-proximal pausing, we calculated the Pol II pausing index, defined as the ratio of promoter to gene body Pol II signal^50,51^, for each gene group across HSCs and their corresponding lineage-committed cells (i.e., MEP genes in MEPs, GMP genes in GMPs, and CLP genes in CLPs). Genes that gained Pol II over the gene body exhibited high pausing indices in HSCs, followed by a significant reduction during differentiation, consistent with a transition to productive elongation (Fig. 6C). Based on these observations, we defined this group as pause-and-release (P&R) genes. In contrast, genes that gain Pol II over the promoter started with low Pol II pausing indices in HSCs, with only a minor, non-significant increase during lineage commitment, suggesting a distinct initiate-and-release (I&R) mechanism (Fig. 6C). By the lineage committed MEP, GMP and CLP stages, both pause-and-release and initiate-and-release genes converged to similarly low pausing indices, likely reflecting the transcriptionally active state of both gene groups in differentiated cells.

Next, we examined how alterations in the chromatin landscape contribute to the transcriptional activation of these gene groups. Previous studies have shown that Pol II can co-occupy promoters with PcG proteins to poise genes for subsequent activation during development^2,15^, but how this co-occupancy relates to Pol II pausing and gene activation remains unclear. In HSCs, we found H3K27me3 was absent from the pause-and-release genes and was significantly more enriched over the initiate-and-release genes (Fig. 6D). This indicates developmental Pol II pausing can be maintained independently of Polycomb-mediated repression.

Surprisingly, H3K27me3 levels over initiate-and-release genes remained elevated even after lineage commitment, suggesting this level of H3K27me3 may dampen, but not entirely prevent transcription. In support of this model, we found the HOX genes, which are deeply repressed by PcG proteins, are marked by significantly higher levels of H3K27me3 than the initiate-and-release genes (Fig. 6D), indicating initiate-and-release genes exist in an intermediate H3K27me3 repressed state.

In contrast to H3K27me3, the H3K4me1-2-3 levels showed dynamic and lineage-specific changes during differentiation. Both pause-and-release, and initiate-and release genes had a significant increase in H3K4me1-2-3 over their promoters in their respective activated lineages (Fig. 6E), and the increase in H3K4me1-2-3 over the gene bodies of pause-and-release genes was also significant (Fig. 6F). These findings suggest that transcriptional activation during lineage commitment is primarily driven by changes in the active chromatin landscape, as reflected by gains in H3K4 methylation.

To determine the relative kinetics of gene activation along the MEP, GMP and CLP lineages, we performed “pseudotime” analysis of mRNA expression using Palantir^52^ (Fig. 6G). For each lineage, pseudotime was normalized from 0 in HSCs to 1 in the most differentiated cells, enabling a unified comparison across trajectories. We then determined the average expression profile of the pause-and-release and initiate-and-release gene groups by plotting the mean z-scaled expression of each gene across it’s respective lineage (Fig. 6H). This analysis revealed a divergence in transcriptional dynamics where pause-and-release genes undergo a rapid burst of transcriptional activation and are then downregulated, whereas initiate-and-release genes are activated more gradually during later stages of differentiation (Fig. 6H). Importantly, this difference in activation timing between the two groups was statistically significant early in pseudotime, supporting a model that in human HSCs Pol II pausing serves as a mechanism for rapid, coordinated transcriptional activation during early lineage commitment, while the Pol II initiate-and-release mechanisms is used to activate genes which are limited by Polycomb repression during a second wave of gene expression (Fig. 6I). Together, these data establish sciCUT&Tag2in1 as a powerful tool for dissecting the temporal and mechanistic logic of transcriptional regulation in primary human samples.

## Discussion:

Our study defines the temporal logic by which adult stem cell activate transcriptional programs in response to environmental stimuli and integrate this with developmentally programmed lineage commitment. We show that Pol II pausing and initiation operate in a sequential cascade, enabling HSCs to mount fast, flexible responses that are re-enforced by slower lineage-commitment programs. These findings help resolve long-standing questions about the functional role of paused Pol II in development and provide a framework for understanding the transcriptional flexibility of human stem cell populations.

To achieve this, we developed sciCUT&Tag2in1, a high specificity, high-yield, and scalable method for joint profiling of Pol II and histone modifications in single cells. Previous extensions of CUT&RUN and CUT&Tag have achieved joint profiling of multiple histone modifications or chromatin-regulatory proteins such as Pol II^23–27,53–59^. However, these methods struggled to produce novel biological insight and require continued technical innovations to address the sparse signals, complex workflows, and limited scalability. Our sciCUT&Tag2in1 method addresses these challenges by leveraging iterative rounds of cellular barcoding to capture Pol II occupancy first, and the more abundant histone marks second, maximizing data recovery while minimizing cross-reactivity. The inclusion of split-barcode reads further boosts Pol II signal, enabling high-quality joint profiling at the scale of tens of thousands of cells per experiment. In addition, the sciCUT&Tag2in1 workflow is highly robust. By lightly cross-linking cells to magnetic beads, we prevent sample loss, and our optimized chromatin release buffer facilitates profiling of whole cells. Also, sciCUT&Tag2in1 uses the same pA-Tn5 protein to profile both Pol II and histone marks, avoiding the need to obtain and purify additional nanobody fusion constructs, and circumventing chemical conjugation of the Tn5 adapter complexes to antibodies that can result in batch-to-batch variability. Together, these features make sciCUT&Tag2in1 particularly well-suited for profiling precious primary human samples where cell numbers are limited. A detailed technical comparison of sciCUT&Tag2in1 to other single-cell multifactorial profiling methods is provided in Supplementary Table 3.

To annotate hematopoietic cell types during single-cell profiling of chromatin-bound proteins, previous studies required either prior cell sorting or multimodal profiling that paired sparse chromatin profiles with more abundant cell-surface markers^28,42^. We demonstrate that single-cell profiling of histone modifications alone can accurately resolve hematopoietic lineage hierarchies. Our results support a previously proposed model that activation-associated histone marks delineate lineage branches, whereas repressive histone marks distinguish HSCs from their differentiated progeny^42^. However, by simultaneously profiling Pol II, sciCUT&Tag2in1 reveals an additional axis of regulatory complexity, capturing cell state transitions associated with cytokine signaling, S-phase cycling, and the paused versus elongating forms of Pol II.

The HSCs we profiled were mobilized by G-CSF, which induces inflammatory stress and likely accounts for the activation of IEGs and increased S-phase index that we observe in HSCs relative to the other progenitor cell types we profiled. By applying computational modelling to single-cell RNA-seq datasets, several studies have identified gene expression signatures indicative of the G1/S and G2/M transitions^60,61^. However, because the histone genes lack a 3’ poly-A tail, they are not detected in standard single-cell RNA-seq pipelines, obscuring the identification of S-Phase cells. The development of similar analysis pipelines to analyze single-cell profiles of Pol II occupancy will enable additional cell-cycle phases to be assigned for quantitative comparisons across heterogenous samples. In tumor samples, Pol II enrichment over the histone genes is correlated with tumor aggressiveness^62^, suggesting that single-cell readouts of the Pol II occupancy over the histone clusters will provide a sensitive readout of the proliferative potential of specific cancer cell types in therapeutic contexts.

Early studies showed that Pol II pausing facilitates rapid gene activation in response to environmental stressors such as heat shock or inflammatory signaling^3,63^. In *Drosophila* embryos Pol II pausing also regulates developmental gene regulation, including the synchronous activation of genes during zygotic genome activation^64^. However, whether paused Pol II serves a kinetic function for rapid activation of developmental gene expression during cell-fate transitions has remained controversial. By leveraging the benefits of single-cell multifactorial profiling, we provide evidence that in human HSCs, Pol II is paused on numerous lineage-commitment genes and serves a kinetic function to enable rapid activation. The selective activation of the pause-release step may also confer regulatory flexibility to HSCs, allowing them to modify their lineage outputs in response to fluctuating physiological demands.

Inhibition of the CDK9/P-TEFb kinase that phosphorylates the Pol II CTD to trigger pause release results in a global suppression of transcription, indicating that most if not all genes include a pausing step in their transcriptional cycle^65^. Our results are consistent with a model that Pol II pausing serves as the developmentally controlled rate-limiting step for the pause-and-release genes. However, Pol II most likely also transiently pauses on the initiate-and-release genes. A recent study identified a rare point mutation in SUPT5H, a component of the DSIF elongation complex, that disrupts erythroid differentiation in humans, and results in decreased expression of *GATA1* and *KLF1*^66^, two genes that we identified in the initiate-and-release group. We find the pausing index for the initiate-and-release genes is low, suggesting pausing is not the rate limiting step that controls the timing of their activation during development. Alternatively, single-cell transcriptomics indicate that transcriptional bursting serves as the rate-limiting step for some developmental genes^67^. Thus, transcriptional burst initiation, rather than Pol II recruitment per se, may serve as the axis of developmental control for the initiate-and-release genes.

Co-occupancy of Pol II and H3K27me3 on gene promoters is referred to as the “balanced” or “poised” state, and marks *Drosophila* patterning genes characterized by highly dynamic expression patterns^2,68^. Although we did not observe the polycomb-associated H3K27me3 mark over the pause-and-release genes in HSCs, we find that in other contexts Polycomb-mediated repression does interact with Pol II to limit transcription. For example, H3K27me3 accumulates over the self-renewal genes *MECOM* and *PRDM16* during HSC differentiation, and although Pol II remains present on the promoter, this coincides with reduction in Pol II elongation into the gene body. In addition, we find that H3K27me3 marks numerous IEGs in the human CD34+ compartment and likely acts to limit stimulus-dependent transcription. This mechanism is evolutionarily conserved, and has also been observed during mouse development, where H3K27me3 is functionally required to dampen transcription of the IEGs^22^. Together, our findings clarify that Pol II pausing in HSCs can be maintained independently of Polycomb repression and show that on other genes Polycomb acts in concert with Pol II to modulate transcriptional output during differentiation.

The sciCUT&Tag2in1 method also opens new avenues for studying the mechanisms controlling HSC inflammatory memory. Numerous lines of evidence support a “top-down” model whereby repeated inflammatory signals are integrated by HSCs to cause sustained myeloid-biased differentiation trajectories of their progeny^16,69,70^. In response to G-CSF-induced inflammatory stress, we observe strong Pol II occupancy over the IEGs in mobilized HSCs, and this is associated with reduced H3K27me3. Notably, In cell culture models of transcriptional memory, repeated rounds of immune stimulation results in elevated transcriptional responses through a progressive depletion of H3K27me3 over the GBP genes^71^. Given the role of Polycomb in propagating gene-regulatory states across DNA replication^10^, it is tempting to speculate that Polycomb-mediated repression may record the number of immune signals HSCs experience. Activation of IEGs during mouse hematopoiesis promotes a myeloid lineage bias^70,72^, and by tuning IEG expression, PcG proteins may influence lineage outcomes of HSC progeny.

The dysregulation of IEGs during HSC immune responses may have direct relevance to human disease. Mutations in Polycomb group genes, including ASXL1 and EZH2, are among the most frequently recurring lesions in myeloproliferative neoplasms (MPNs) and myelodysplastic syndromes (MDS)^18–21^. Emerging evidence suggests these mutations, along with others affecting splicing, DNA methylation, and cytokine signaling, converge on hyperactivation of innate immune genes^17,73^. Our finding that Polycomb normally represses IEGs in HSCs points to a model in which loss of this repression, whether by inflammation, mutation, or both, may contribute to pathologically enhanced inflammatory signaling and fuel clonal evolution during MPN and MDS. Future studies dissecting the specific Polycomb complexes involved in inflammatory memory and hematologic malignancy will be critical.

Together, our findings position sciCUT&Tag2in1 as a transformative platform for dissecting the interface of transcriptional regulation and epigenetic programs in complex biological systems and provide a foundation for studying stem cell inflammatory memory, and the evolution of myeloid malignancies at single-cell resolution.

### Limitations of the Study:

While sciCUT&Tag2in1 represents a major technical advance, limitations remain. Joint profiling of Pol II requires tagmentation under CUTAC conditions to prevent cross-reactivity, and currently the split-barcode reads are enriched over Pol II sites, but do not necessarily represent individual loci that are co-occupied by Pol II and histone marks. Thus, additional technical innovation is necessary to achieve single-molecule resolution of protein co-occupancy using CUT&Tag-based approaches. In addition, QC filtering may exclude low-signal populations, for example monocytes, which have inherently low global H3K27me3^74^. Future applications may benefit from direct pairing of complementary marks, such as H3K4me1-2-3 and H3K27me3, as this may help recover cell types with low abundance of one mark or the other, improving sensitivity for rare or epigenetically distinct populations.

### Resource availability:

#### Lead Contact

Questions related to this study and requests for resources and reagents should be directed to the lead contact, Derek Janssens (Derek.Janssens@vai.org), and will be fulfilled in a timely manner.

#### Materials Availability

Materials generated in this study will be available upon reasonable request.

## STAR Methods:

### EXPERIMENTAL MODEL AND STUDY PARTICIPANT DETAILS

#### CD34+ Sample Information

The CD34+ samples were obtained by the Fred Hutchinson Cooperative Center for Excellence in Hematology (CCEH) from volunteer donors in accordance with the Declaration of Helsinki. This involved administration of G-CSF to mobilize the HSCs and progenitors from the bone marrow, collection of peripheral blood and enrichment with anti-CD34+ antibody-conjugated magnetic beads. Written consent was obtained from all patients to permit the use of their de-identified samples in medical research. For four patient samples there was not explicit consent for sharing primary DNA sequencing data and, because all data was collected using donor mixing experiments that typically included one of these four donors, to protect any patient-specific sequence information the data is provided as hg38 aligned files only. The studies were overseen by the institutional review boards at the Fred Hutch Cancer Center (IR protocol 9950).

The CD34+ samples were de-identified, but to ensure the data collected as part of this study were broadly representative of the human population, we included an equal proportion of male and female donors, as well as donors that self-reported their race as white, black and Asian and ranged in age from 22-56 years old. Donors were compensated by the CCEH for undergoing the G-CSF mobilization and blood draw procedures, but this was in no way motivated or related to inclusion of their samples in this study.

### METHOD DETAILS

#### Antibodies

For profiling Polycomb-mediated repression, we used the rabbit monoclonal anti-H3K27me3 antibody (Cell Signaling Technologies, cat. no. 9733; RRID: AB_2616029). For profiling the TrxG-protein regulome, we used three antibodies: rabbit oligoclonal anti-H3K4me1 (Thermo, cat. no. 710795; RRID: AB_2532764), rabbit polyclonal anti-H3K4me2 (Millipore, cat. no. 07-030; RRID: AB_310342) and rabbit polyclonal anti-H3K4me3 (Active Motif, cat. no. 39159; RRID: AB_2615077). For profiling both the paused and elongating forms of Pol II, we used a combination of three antibodies: rabbit monoclonal anti-Phospho-Ser5-Rpb1 CTD (Cell Signaling Technologies; cat. no. 13523; RRID: AB_2798246), rabbit monoclonal anti-Phospho-Ser2-Rpb1 CTD (Cell Signaling Technologies; cat. no. 13499; RRID: AB_2798238) and rabbit monoclonal anti-Phospho-Ser2/Ser5-Rpb1 CTD (Cell Signaling Technologies; cat. no. 13546; RRID: AB_2798253). All reactions also included a subsequent incubation with the secondary guinea pig anti-rabbit IgG antibody (Antibodies Online, cat. no. ABIN101961, RRID: AB_10775589), this secondary serves as an adaptor to amplify the number of tethered pA-Tn5 complexes.

#### Optimization of sciCUT&Tag for profiling whole cells

To determine the optimal conditions for sciCUT&Tag profiling of whole cells, we prepared 1 million CD34+ nuclei and 1 million CD34+ whole cells in parallel. Whole cells were spun down at 600 X G and brought up in 500 μL Wash Buffer containing Triton-X 100 for permeabilization (48 mL ddH_2_O, 1 mL 1M HEPES pH 7.5 [20 mM], 1.5 mL 5M NaCl [150 mM], 12.5 μL 2M Spermidine [0.5 mM], 250 μL of 10% Triton-X-100 [0.05%],1 Roche Complete EDTA-free tablet). MAGNEFY ConA magnetic beads (Bangs, cat. no. MFY531) were prepared by washing 200 μL, 2 times with 500 μL Binding Buffer (400 μL of 1 M HEPES-KOH pH 7.9 [20 mM], 200 μL of 1 M KCl [10 mM] 20 μL of 1 M CaCl_2_ [1 mM], and 20 μL of 1 M MnCl_2_ [1 mM], bring up to 20 mL with nanopure water). MAGNEFY ConA Beads were resuspended in 200 μL of Binding Buffer, and 100 μL of beads were added to the nuclei and whole cell suspensions in 3-33 μL increments, the sample was mixed by inversion after each addition. Bead binding was allowed to proceed for 10 min at room temperature, and the supernatant was removed on a magnet stand, and nuclei were resuspended in 60 uL of Antibody Buffer (2 mL Wash Buffer with 8 μL 0.5M EDTA [2 mM]). Whole cells were brought up in 500 μL of Wash Buffer and lightly crosslinked to prevent lysis and aggregation throughout the protocol, by adding 3.2 μL of 16% formaldehyde (Fischer Scientific, cat. no. 28906). Light cross-linking is necessary to prevent cell lysis and aggregation during single-cell CUT&Tag^74^, and previous comparisons made in bulk demonstrated that CUT&Tag signal generated in native and cross-linked cells was indistinguishable by genome-wide correlations as well as FRiP analysis^23^. The cross-linking reaction was quenched after 2 min by adding 15 μL of 2.5 M glycine and then washing cells once with 500 μL of Wash Buffer and bringing them up in 60 μL of Antibody Buffer. Cells and nuclei were then split into two 30 μL samples and incubated overnight with either 3 μL of H3K27me3 antibody or 3 μL of H3K4me1, 3 μL of H3K4me2 and 3 μL of the H3K4me3 antibody.

The next day, the sciCUT&Tag protocol was carried out as previously described^74^, with the following exceptions: (1) following guinea-pig anti-rabbit secondary binding, the H3K4me1-2-3 nuclei and whole cells and H3K27me3 nuclei and whole cells were each arrayed in 24 wells of a 96 well plate (Eppendorf, cat. no. 0030129504), and (2) 12 wells of each were incubated with either the sci-pA-Tn5 complexes loaded with adapters purified by standard desalting conditions, and 12 wells of each were incubated with sci-pA-Tn5 complexes loaded with PAGE purified adapters. Cells were washed, tagmented, pooled and passed through a 20-micron filter and dispensed on an ICELL8 nanowell dispenser into a 350v ICELL8 chip according to the standard protocol. (3) We then loaded 4 wells of the ICELL8 source plate with standard Chromatin Release Buffer (648 μL 10 mM TAPS, 152 μL 1% SDS) and we loaded the other 4 wells of the ICELL8 source plate with Chromatin Release Buffer containing thermolabile proteinase K (616 μL 10 mM TAPS, 152 μL 1% SDS, 32 μL Thermolabile Proteinase K). (4) After the 35 nL dispense, the chip was sealed with thermal film, spun down and incubated at 37 °C for 1 hour to allow proteinase K digestion, and 58 °C for 1 hour to denature Thermolabile Proteinase K and other chromatin proteins. The rest of the experiment was carried out according to the standard protocol. This experiment demonstrated that loading pA-Tn5 with PAGE purified oligos and including Thermolabile Proteinase K in the Chromatin Release Buffer increases the number of reads recovered per cell, as well as the signal-to-noise as measured by the Fraction of Reads in Peaks.

#### sciCUT&Tag2in1 Procedure

Prior to starting the experiment, two 96-well plates containing differentially barcoded pA-Tn5 complexes were prepared as described previously^74^. For sciCUT&Tag2in1 we generated a second sci-pA-Tn5 plate loaded with uniquely barcoded DNA-adapters that were synthesized as single-stranded oligos, PAGE purified, annealed to the reverse adapter (Tn5MErev) and used to load pA-Tn5 purified in house. The sequences of all oligos used for sciCUT&Tag2in1 are listed in Supplementary Table 1.

Whole CD34^+^ cells from up to eight donors were thawed and mixed to a final count of 2 million cells. MAGNEFY ConA beads (Bangs, cat. no. MFY531) were used for cell binding, and magnetic separation during wash steps, and profiling was performed first for Pol2-Ser5P, Pol2-Ser2/5P, and Pol2-Ser2P using the CUTAC approach with 96 differentially barcoded pA-Tn5 complexes, followed by profiling for H3K4me1-2-3 or H3K27me3 in a second round of tagmentation under normal CUT&Tag conditions, using a second set of 96 differentially barcoded pA-Tn5 complexes.

On Day 1, whole CD34^+^ cells from up to eight donors were thawed at 37 °C, and mixed to a final count of 2 million cells. Excess cells that were not used in the experiment were aliquoted and refrozen at −80 °C in a Mr. Frosty Isopropanol Chamber for future use. Cells were pooled and pelleted at 600 X g for 3 min, and brought up in 900 μL of Wash Buffer (48 mL ddH_2_O, 1 mL 1M HEPES pH 7.5 [20 mM], 1.5 mL 5M NaCl [150 mM], 12.5 μL 2M Spermidine [0.5 mM], 250 μL of 10% Triton-X-100 [0.05%],1 Roche Complete EDTA-free tablet). To prepare magnetic beads for binding cells, 200 μL of MAGNEFY ConA beads were washed 2 times on a magnet stand with 500 μL Binding Buffer (400 μL of 1 M HEPES-KOH pH 7.9 [20 mM], 200 μL of 1 M KCl [10 mM] 20 μL of 1 M CaCl_2_ [1 mM], and 20 μL of 1 M MnCl_2_ [1 mM], bring up to 20 mL with nanopure water). Beads were resuspended in 100 μL Binding Buffer and added to the whole-cell suspension in 3-33 μL increments, the sample was mixed by inversion after each addition. Bead binding was allowed to proceed for 10 min at room temperature, and the supernatant was removed on a magnet stand. Whole cells were brought up in 500 μL of Wash Buffer and lightly crosslinked by adding 3.2 μL of 16% formaldehyde (Fischer Scientific, cat. no. 28906), for a final concentration of 0.1 % formaldehyde during fixation. The reaction was quenched after 2 min by adding 15 μL of 2.5 M glycine and washing cells with 500 μL of Wash Buffer and bringing them up in 50 μL of Antibody Buffer (2 mL Wash Buffer with 8 μL 0.5M EDTA [2 mM]). Cells were then incubated overnight with 5 μL of anti-Phospho-Ser5-Rpb1 CTD, 5 μL of anti-Phospho-Ser2-Rpb1 CT antibody and 5 μL of anti-Phospho-Ser2/5-Rpb1 CTD antibodies.

On Day 2, 300-Wash Buffer was prepared (48 mL ddH_2_O, 1 mL 1M HEPES pH 7.5 [20 mM], 3 mL 5M NaCl [300 mM], 12.5 μL 2M Spermidine [0.5 mM], 1 Roche Complete EDTA-free tablet). TAPS Buffer was also prepared (500 μL 1M TAPS pH 8.5 [10 mM] in 49.5 mL ddH_2_O).Samples were washed 2 times with 100 μL Wash Buffer, resuspended in 30 μL Wash Buffer, and 2 μL of secondary antibody (Guinea Pig anti-Rabbit) was added for 1 hour at 4 °C. Cells were washed again, resuspended in 1500 μL of 300-Wash Buffer, and 15 uL of sample was distributed into each well of a 96-well plate (Eppendorf, cat. no. 0030129504). 1.5 μL of the differentially barcoded pA-Tn5 complexes from sci-pA-Tn5 plate 1 was added to each of the samples, and the plate was sealed and incubated for 1 hour at 4 °C. Cells were washed on a 96-ring low elution magnet (ALPAQU cat. no. A000350) two times with 50 μL of 300-Wash Buffer. Cells were brought up in 50 μL of CUTAC Tagmentation buffer (10 mL Wash Buffer + 100 μL 1M MgCl_2_ [10 mM]), and tagmentation was carried out at 37 °C for 30 min on a thermocycler with a heated lid. Cells were then washed two times on the magnet stand with 100 μL Wash Buffer and resuspended in 9.5 μL of the Rb-Fc Block solution (1 mL Wash Buffer + 20 μL Rabbit-Fc-Peptide at 1 mg/mL; Rockland, cat. no. 011-0103). Rabbit-Fc Block minimizes cross-reactivity of the second round of antibodies with the residual Guinea-Pig anti-rabbit secondary binding sites that are present on the chromatin surrounding the Pol II target. The blocking step was carried out at 4 °C for 1 hour.

H3K4me1-2-3 antibody mix (Antibody solution-1) was prepared with 375 μL Wash Buffer + 1.5 μL 0.5M EDTA [2 mM] + 20 μL each of H3K4me1, H3K4me2, and H3K4me3. H3K27me3 antibody mix (Antibody solution-2) was prepared with 375 μL Wash Buffer + 1.5 μL 0.5M EDTA [2 mM] + 30 μL H3K27me3. Cells were washed twice on a 96-ring low elution magnet with 50 μL of Wash Buffer, and 48 wells were brought up in 7.25 μL of Antibody solution-1 and 48 wells were brought up in 7.25 μL of Antibody solution-2 and incubated at room temperature for 1 hour. Secondary antibody solution-2 was made by mixing 1 mL Wash Buffer with 35 μL Guinea Pig anti-Rabbit. After washing two times with 50 μL of Wash Buffer on the magnet, cells were brought up in 9.5 μL of the secondary antibody mix and incubated for 1 hour at 4 °C. Cells were washed two times with 50 μL of Wash Buffer on the magnet, brought up in 15 μL 300-Wash Buffer, and 1.5 μL of the differentially barcoded pA-Tn5 complexes from sci-pA-Tn5 plate 2 were added, and allowed to bind by sealing the plate and incubating for 1 hour at 4 °C. CUT&Tag Tagmentation Buffer was prepared (10 mL 300-Wash Buffer + 100 μL 1M MgCl_2_ [10 mM]). After washing two times with 50 μL of 300-Wash, cells were resuspended in 50 μL Tagmentation Buffer, and tagmentation was carried out at 37 °C for 1 hour on a thermocycler with a heated lid. Tagmentation Buffer was removed on the magnet stand, and all wells were washed with 100 μL Wash Buffer and pooled into a 0.6 mL LoBind Tube using an 8-channel pipette and wide-bore tips. A second 100 μL of Wash Buffer was used to capture residual material, and this was added to the pool in the 0.6 mL LoBind Tube. Wash Buffer was removed, and cells were brought up in 540 μL of Wash Buffer.

For size filtering, 270 μL of the pooled sample was passed through 2-20 μm filters (PluriSelect USA, cat. no. 43-10020-40) at 100 × g for 3 min, and flow-through was retained and combined into a single sample again. DAPI staining was performed by adding 10 μL of 10,000× DAPI diluted 2:198 in TAPS Buffer. Nuclei were counted on a Countess slide and imaged. Immediately before loading the ICELL8 source wells for dispensing into and ICELL8 350v chip, the sample was diluted to 10 cells per 35 nL in 10 mM TAPS, and DAPI solution was added for a final concentration of 1X. Then 65 μL of the cell suspension was loaded into wells A1–D2 on the ICELL8 source plate, and 0.19% SDS Release Buffer was prepared (616 μL 10 mM TAPS, 152 μL 1% SDS, 32 μL Thermolabile Proteinase K).

iCell8 dispensing involved: 35 nL of nuclei on beads (dispense one); drying, sealing, spinning at 1,200 X g for 1 min and imaging; 35 nL of 0.19% SDS + thermolabile proteinase K in TAPS (dispense two); drying, heat sealing, spinning, cooking at 37 °C for 1 h and then 58 °C for 1 h, cooling at 4 °C overnight; spinning, 35 nL of 2.5% TritonX-100 (dispense three, prepared as 250 μL 10% TritonX-100 in 750 μL ddH_2_O); drying, sealing, spinning, 35 nL i5 primer (dispense four); drying, sealing, spinning, 35 nL i7 primer (dispense five), followed by drying, sealing and spinning. KAPA PCR Mix was prepared with 553.7 μL 5 X HiFi buffer, 306.6 μL ddH_2_O, 84.8 μL 10 mM dNTP, 54.9 μL KAPA HiFi polymerase. Then, dispensing on the ICELL8 was resumed with 50 nL of KAPA PCR Mix (dispense six), followed by drying, sealing, spinning, and a second 50 nL more KAPA Mix (dispense seven). The chip was heat sealed, spun, and run through a PCR profile with: 58 °C for 5 min (gap fill), 72 °C for 5 min, 98 °C for 45 sec, followed by 13 cycles of (98 °C for 15 s, 60 °C for 10 s, 72 °C for 5 s), ending with 72 °C for 1 min and hold at 8 °C. After amplification, 1.3 X Ampure bead cleanup was performed, and DNA was resuspended in 100 μL Tris-HCl pH 8, a second 0.9 X Ampure bead cleanup was performed, and DNA was resuspended in 100 μL Tris-HCl pH 8. Final libraries were analyzed on a TapeStation.

#### DNA sequencing and data processing

sciCUT&Tag and sciCUT&Tag2in1 libraries were sequenced on an illumina NextSeq P2-300 flow cell using the custom primers NextSeq_Read1_seq_primer, NextSeq_Read2_seq_primer, NextSeq_Index1_seq_primer and NextSeq_Index2_seq_primer (Supplementary Table 1). The read lengths were as follows: Read 1 = 79 bp, Index 1 = 43 bp, Index 2 = 37 bp, and Read 2= 79 bp. Demultiplexing was performed using our previously generated sciCTextract software (https://github.com/mfitzgib/sciCTextract)^74^. Adapter trimming was performed using cutadapt 2.9 with parameters: -j 8 -m 20 -a CTGTCTCTTATACACATCT -ACTGTCTCTTATACACATCT -Z, and Fastq files were aligned to the hg38 genome using Bowtie2 version 2.4.2 with the parameters: –very-sensitive-local –soft-clipped-unmapped-tlen –no-mixed –no-discordant –dovetail –phred33 -I 10 -X 1000. For each experiment, cells from 1 to 8 donors were pooled, and SNP-based donor identification and doublet detection was performed using Souporcell^32^. To accommodate the large dataset, we performed souporcell on three separate batches, and we used the aligned SAM files as input to our previously generated Souper-star pipeline^74^. To match donor calls between Souporcell runs, we included the control H3K4me1-2-3 datasets that contained only a single donor in all three runs. The paired Pol II, H3K4me1-2-3, H3K27me3, and split-barcode datasets were all processed independently, and to facilitate easy cross-comparisons between paired datasets, the barcodes in the H3K4me1-2-3, H3K27me3 and split-read barcodes were all rewritten in the aligned bed files to match the paired Pol II data.

#### SciCUT&Tag2in1 QC and data analysis

We began our sciCUT&Tag2in1 data analysis by generating a cell-by-genomic-bins count matrix for each dataset using a bin size of 5kb for H3K4me1-2-3 and 50kb for H3K27me3 and Pol II. For dimensionality reduction we used the addIterativeLSI() function in ArchR with the parameters: iterations = 2, dimstoUse = 1:15, varFeatures = 20,0000, clusterParams = list(resolution = c(0.1,0.3), sampleCells = 8000, n.start = 10), outlierQuantiles = c(0.025, 0.975), verbose = FALSE, force = TRUE. To organize cells in UMAP space, we first performed batch correction using the addHarmony() function in archR with the parameters: reducedDims = “IterativeLSI”, name = “Harmony”, groupBy = “Sample”, and then applied the addUMAP() function with the parameters: reducedDims = “Harmony”, nNeighbors = 40, minDist = 0.4, metric = “cosine”. For H3K27me3 UMAPs, sex chromosome reads were excluded to avoid donor separation driven by X-inactivation.

We then filtered cells using the following thresholds: for H3K4me1-2-3, H3K27me3 and Pol II we kept cells with ≥250 unique reads, for H3K4me1-2-3 we applied cutoffs of FRiP > 0.75, FRiB < 0.075, for H3K27me3 we identified two clusters with reduced FRiPs and elevated FRiBs and these clusters were removed, for Pol II we identified one cluster with reduced FRiPs and we kept cells with FRiP > 0.50 and one cluster with elevated FRiPs and we kept cells with FRiP < 0.775.

For doublet removal we used iterative rounds of UMAP embedding followed by high-resolution clustering using the addClusters() function in ArchR with the parameters: resolution = 20, maxClusters = 100, and removal cluster enriched for Souporcell-defined doublets.

For H3K4me1-2-3, we identified 95 features driving artificial clustering (e.g., FAM72A-D, SRGAP2A-C, rDNA) and these features were added to the ArchR backlist and were excluded during subsequent ArchR analysis.

For identification of cluster-specific marker genes we performed low-resolution clustering using the addClusters() function in ArchR with the parameters: resolution = 2, maxClusters = 30, and then manually combined clusters from neighboring regions in the UMAP. We then calculated the gene scores using the addGeneScoreMatrix() function and used the addImputeWeights() function to impute the gene scores in surrounding cells. We then used the getMarkerFeatures() function with the parameters: bias = c(“TSSEnrichment”, “log10(nFrags)”), testMethod = “wilcoxon”, and called all genes with an FDR <=0.05 and Log2 Fold Change >= 1 and Log2 Fold Change <=−1 (Supplementary Table 2).

#### Comparisons to RNA-seq

To examine the expression patterns of cluster-specific marker genes, we overlaid ArchR-derived marker lists onto a previously generated UMAP embedding of single-cell RNA-seq profiles from lineage-depleted bone marrow^36^. We calculated the average z-scores for each cluster using the genes-expressed in at least 50 cells in the RNA-seq dataset.

For the H3K4me1-2-3 clusters, we applied a log_2_ fold change threshold > 1. In the HSC cluster, 44 marker genes passed this threshold, of which 21 were detected above the RNA-seq expression threshold. In the MEP cluster, 34 genes passed the marker threshold (FDR < 1 × 10^−4^), and 23 were detected in the RNA-seq data. In the GMP cluster, 3 marker genes were identified (FDR < 1 × 10^−4^), 2 of which were expressed by RNA-seq. The CLP cluster yielded 47 marker genes (FDR < 1 × 10^−4^), and 29 were expressed. The Pre-B Cell cluster contained 385 markers (FDR < 1 × 10^−4^), of which 208 were detected. For the B Cell cluster, 539 genes were identified (FDR < 6 × 10^−5^), with 388 above the RNA-seq detection threshold. Finally, in the Monocyte cluster, 371 marker genes were identified (FDR < 1 × 10^−4^), with 289 detected by RNA-seq.

For the H3K27me3 clusters, we applied a log_2_ fold change threshold < −1. In the HSC cluster, 75 marker genes were identified (FDR < 1 × 10^−4^), 47 of which were detected by RNA-seq. The MEP cluster yielded 48 markers (FDR < 0.05), with 17 expressed above the threshold. In the PBMC1 and PBMC2 clusters, we identified 226 and 586 markers, respectively (FDR < 1 × 10^−4^ and 1 × 10^−10^), of which 84 and 220 were detected in the RNA-seq dataset.

Pseudotime analysis was performed to examine the pause-and-release, versus initiate-and-release genes along the MEP, GMP and CLP lineage trajectories of the RNA-seq UMAP as previously described^36,52^.

#### Defining Gene Promoters and Gene Bodies

To define promoter and gene body intervals for CUT&Tag analysis, we first parsed the GENCODE v38 GTF annotation to extract transcript-level information and transcription start sites (TSS). Strand-specific ±3 kb windows were generated for each transcript to define initial promoter intervals. These were intersected with Pol II data using *pybedtools*, and quantified as normalized signal density. For genes with multiple annotated transcripts, we retained the promoter interval with the highest normalized CUTAC signal. Promoter size was then scaled based on gene length to minimize overlap with gene bodies: for genes >30 kb, the original ±3 kb window (6 kb total) was retained; for genes 20–30 kb in length, promoters were reduced to ±2 kb (4 kb total); for genes 10–20 kb, to ±1.5 kb (3 kb total); and for genes <10 kb, to ±1 kb (2 kb total). These size-adjusted promoter intervals were merged with strand-specific ArchR gene annotations to define gene body regions, ensuring non-overlapping start and end points. Implausibly large genes (>2.5 Mb) were excluded. Final promoter and gene body BED files were used for downstream analyses of Pol II and histone modifications.

#### Cluster-specific enrichment of Pol II

To identify cluster-enriched Pol II binding sites, we first quantified the number of overlapping reads for each candidate genomic interval and the single-cell Pol II profiles. For each cell cluster of interest, we separately tallied reads originating from that cluster (cluster-specific reads) and from all other HSPCs excluding the cluster (background HSPCs). To evaluate enrichment, we employed a Bayesian framework in which these counts were modeled using independent multinomial distributions with uniform Dirichlet priors, reflecting no prior assumptions about the distribution of reads across intervals.

For each interval, the posterior distributions of the read proportions assigned to the cluster-specific reads and background HSPC sets were estimated. Specifically, for each condition, the count vector across genomic intervals was incremented by one and treated as a Dirichlet parameter, yielding a posterior probability for the fraction of reads assigned to each region. The log2 ratio of the cluster-specific to background read proportions was then computed and referred to as the cluster Pol II enrichment score.

To estimate the uncertainty of this score, we performed Monte Carlo sampling (N = 1000) from the posterior distributions to derive the mean and standard deviation of the log-ratio for each interval. Using these, we calculated a z-score and corresponding p-value for each site. Multiple hypothesis correction was performed using the Benjamini–Hochberg method as implemented in the statsmodels Python package. This strategy was applied to the promoter intervals and the gene body intervals separately, and then the two values were combined for each gene.

### QUANTIFICATION AND STATISTICAL ANALYSIS

To compare sciCUT&Tag and sciCUT&Tag2in1 protocol variants, we assessed differences in read count per cell and FRiPs. For the Pol II, H3K4me1-2-3 and H3K27me3 datasets we performed pairwise comparisons between protocol groups using two-sided independent samples t-tests with unequal sample sizes. Because the number of datasets was limited, we did not correct for multiple hypothesis testing.

To evaluate barcode pairing fidelity in sciCUT&Tag2in1 datasets, we compared the fraction of matched reads between paired and mismatched donor combinations for H3K4me1-2-3 and H3K27me3 datasets. For each histone mark, we conducted one-sided independent samples t-tests to test the hypothesis that paired donor samples yield a higher fraction of correctly matched reads than mismatched samples. Because the number of datasets was limited, we did not correct for multiple hypothesis testing.

To evaluate the total read counts between matched and mismatched samples for both H3K4me1-2-3 and H3K27me3 datasets, we performed two-sided independent samples *t*-tests assuming unequal group sizes, with *n* representing the number of single-cell profiles in each group. Because the number of datasets was limited, we did not correct for multiple hypothesis testing.

To assess differences in Pol II occupancy between S-phase and non–S-phase cells, we stratified single cells based on a cutoff of 0.0025 for the fraction of total Pol II reads over histone genes. Cells with values above this threshold were classified as “S_Phase,” and those below as “Non_S_Phase.” For each group, we compared total aligned reads using two-sided Mann–Whitney U tests, a non-parametric alternative appropriate for comparing distributions without assuming normality.

To evaluate differential Pol II and H3K27me3 enrichment across immediate early gene (IEG) loci between HSCs and other hematopoietic populations, we performed one-sided t-tests across manually curated genomic intervals extending form the IEG promoter out to the transcriptional termination site. For each interval, we compared z-scored normalized Pol II, H3K27me3 and H3K4me1-2-3 signal between cells classified as HSCs and those in the differentiated cell group, and single-cells from separate experiments were considered as biological replicates and the signal aggregated for comparison. A one-sided test was used to determine whether Pol II and H3K4me1-2-3 occupancy was significantly higher in HSCs, consistent with activation. And a one-sided test was used to determine whether H3K27me3 occupancy was significantly lower in HSCs, as a lack of repression was expected.

To assess dynamic changes in sciCUT&Tag2in1 signals across the HSC-to-MEP lineage trajectory, we defined a curated list of peak regions (Promoter and DE elements) and quantified signal from the temporally ordered clusters. For each dataset, experiments run separately were considered as biological replicates, and the aggregate BED files were intersected with the fixed peak intervals using pybedtools, and the number of overlapping reads per interval was normalized by total read count to yield per-cluster normalized scores. These values were then z-score normalized within datasets across each interval to facilitate relative comparisons. To statistically evaluate changes along the trajectory, we performed a series of pairwise t-tests between successive MEP stages (e.g., HSC vs MPP early) for each interval using the z-transformed scores. For comparisons involving histone marks we used a two-sided t-test as the directionality of change was not necessarily expected, whereas for temporal comparisons of the Pol II datasets we used a one-sided t-test because in each case we had a clear hypothesis going in regarding which direction the value would change.

To assess differential gene expression across selected cell populations in the scRNA-seq dataset^36^, we performed a series of targeted pairwise comparisons between predefined groups (e.g., HSC vs MEP, GMP vs CLP) using non-parametric statistical testing. For each gene and comparison, a Wilcoxon rank-sum test was applied to quantify differences in expression levels, and the resulting p-values were adjusted for multiple testing using the Benjamini-Hochberg procedure to control the false discovery rate (FDR). Alongside statistical significance, we computed the mean expression, percent of cells expressing the gene, and log fold change between groups to provide a comprehensive view of expression dynamics. These comparisons were restricted to a curated gene list and were repeated independently for each pair of cell types, enabling targeted evaluation of lineage-specific expression programs.

To compare the temporal activation dynamics of pause-and-release (P&R) and initiate-and-release (I&R) gene groups during lineage commitment, we calculated average z-score normalized gene expression across pseudotime windows. For each lineage branch, we used gene expression trends imputed by Palantir and computed the mean z-scaled expression of each gene within defined pseudotime intervals. Genes were grouped by activation class (P&R vs. I&R), and comparisons were pooled across multiple branches (e.g., MEP, GMP, CLP) where the relevant genes were expressed. We then performed Welch’s t-tests (unequal variance) to compare mean expression between the two gene groups within each pseudotime window. This approach enabled a quantitative assessment of whether the P&R and I&R gene groups exhibited significantly different activation kinetics across temporally defined stages of differentiation.

## Supplementary Material

Supplementary Figures

Supplementary Table 1 Table S1. Excel File listing the DNA oligo sequences used for sciCUT&Tag2in1 library generation and sequencing.

Supplementary Table 2 Table S2. Excel File listing the ArchR-imputed H3K4me1-2-3, H3K27me3 and Pol II cluster-specific marker genes.

Supplementary Table 3 Table S3. Excel File comparing sciCUT&Tag2in1 to other multifactorial single-cell profiling methods.

## Figures and Tables

**Table:** 

REAGENT or RESOURCE	SOURCE	IDENTIFIER
Antibodies		
Rabbit monoclonal anti-H3K27me3 antibody	Cell Signaling Technologies	Cat#9733; RRID: AB_2616029
Rabbit oligoclonal anti-H3K4me1	Thermo	Cat#710795; RRID: AB_2532764
Rabbit polyclonal anti-H3K4me2	Millipore	Cat#07-030; RRID: AB_310342
Rabbit polyclonal anti-H3K4me3	Active Motif	Cat#39159; RRID: AB_2615077
Rabbit monoclonal anti-Phospho-Ser5-Rpb1 CTD	Cell Signaling Technologies	Cat#13523; RRID: AB_2798246
Rabbit monoclonal anti-Phospho-Ser2-Rpb1 CTD	Cell Signaling Technologies	Cat#13499; RRID: AB_2798238
Rabbit monoclonal anti-Phospho-Ser2/Ser5-Rpb1 CTD	Cell Signaling Technologies	Cat#13546; RRID: AB_2798253
Guinea pig anti-rabbit IgG secondary	Antibodies Online	Cat#ABIN101961; RRID: AB_10775589
Biological samples		
G-CSF mobilized CD34+ HSPCs	Fred Hutch Cancer Center; Center for Excellence in Hematology	N/A
Chemicals, peptides, and recombinant proteins		
Rabbit-Fc-Peptide	Rockland	Cat#011-0103; RRID: AB_840602
pA-Tn5 loaded with PAGE purified adapters	This study	dx.doi.org/10.17504/protocols.io.8yrhxv6
Critical commercial assays		
ICELL8 instrument	TAKARA	N/A
350v ICELL8 chip	TAKARA	Cat#640019
Single-Cell Loading Kit	TAKARA	Cat#640206
Single-Cell Collection Kit	TAKARA	Cat#640212
MAGNEFY ConA beads	Bangs	Cat#MFY531
Thermolabile Proteinase K	NEB	Cat#P8111L
20 μm filters	PluriSelect USA	Cat#43-10020-40
96-ring low elution magnet	ALPAQUA	Cat#A000350
KAPA HiFi PCR Kit with dNTPs	Roche	Cat#07958838001
NextSeq 2000 sequencing Instrument	Illumina	N/A
NextSeq P2-300 flow cell	Illumina	N/A
Tapestation 4200	Agilent	N/A
High Sensitivity D100 Screen Tape Assay	Agilent	N/A
Deposited data		
sciCUT&Tag of G-CSF mobilized CD34+ HSPCs provided as bed files aligned to hg38	This study; Zenodo	https://doi.org/10.5281/zenodo.17782193
sciCUT&Tag2in1 of G-CSF mobilized CD34+ HSPCs provided as bed files aligned to hg38	This study; Zenodo	https://doi.org/10.5281/zenodo.17782193
T-Cell Depleted Bone Marrow Multiome Data	Persad et al.^77^	https://doi.org/10.5281/zenodo.6383269
Oligonucleotides		
Barcoded adapter oligos (PAGE purified) for loading pA-Tn5, see Table S1	This Study; ordered from IDT	N/A
Barcoded primers for sciCUT&Tag PCR, see Table S1	This Study; ordered from IDT	N/A
Primers for custom sequencing of sciCUT&Tag libraries, see Table S1	This Study; ordered from IDT	N/A
Recombinant DNA		
3XFlag-pA-Tn5-Fl (pA-Tn5) plasmid	Addgene	Cat#124601
Software and algorithms		
sciCTextract	Janssens et al.^74^	https://github.com/mfitzgib/sciCTextract
Cutadapt 2.9	Marcel Martin	DOI:10.14806/ej.17.1.200
Bowtie2 2.4.2	Langmead et al.^78^	https://pypi.org/project/cutadapt/
Souporcell	Heaton et al. ^32^	https://github.com/wheaton5/souporcell
R version 4.4.0 (2024-04-24)	R Core Team	https://www.R-project.org/
ArchR 1 0 2	Granja et al. ^35^	https://github.com/GreenleafLab/ArchR
GenomicRanges 1 58 0	Lawrence et al.^79^	10.1371/journal.pcbi.1003118
Ggplot2 3 5 2	Wickham et al. ^80^	https://ggplot2.tidyverse.org
Seurat 5 3 0	Hao et al. ^81^	https://satijalab.org/seurat/articles/install.html
Python 3.11.3	Python Software Foundation	https://www.python.org
Pandas 2.2.3	PyPl	RRID:SCR_018214
Numpy 1.26.4	PyPl	RRID:SCR_008633
Seaborn 0.13.2	PyPl	RRID:SCR_018132
Pybedtools 0.10.0	PyPl	RRID:SCR_006646
matplotlib	PyPl	RRID:SCR_008624
scipy	PyPl	RRID:SCR_008058
statsmodels	PyPl	RRID:SCR_016074
tqdm	PyPl	RRID:SCR_018442
Palantir 1.4.0	Setty et al.^52^	https://github.com/dpeerlab/Palantir
Other		
96 well plate	Eppendorf	Cat#0030129504

## Data Availability

The aligned and processed sciCUT&Tag2in1 and sciCUT&Tag data generated in this study will are available through the zenodo data sharing portal^75^. During preparation of this manuscript, we were made aware that consent for genomic data sharing was not obtained from four of the ten donors of the CD34+ samples used in this study. All experiments were done using mixtures of cells that typically included at least one of these four donors. Therefore, to protect any patient-specific sequence information the data is provided as hg38 aligned files only, and the primary sequencing data will not be made available. All custom code used for data analysis and figure generation are available through GitHub^76^. Any additional information required to reanalyze the data reported in this study will be made available upon reasonable request made to the lead contact.
