# Supplementary material for: Sequential RNA Polymerase II Activation Drives Human Hematopoiesis
Source: Cell Rep. Author manuscript; Available in PMC 2026 Apr 10. (PMC13067999; doi:10.1016/j.celrep.2025.116802)
Supplement: Supplementary Figures [file NIHMS2137280-supplement-Supplementary_Figures.pdf]

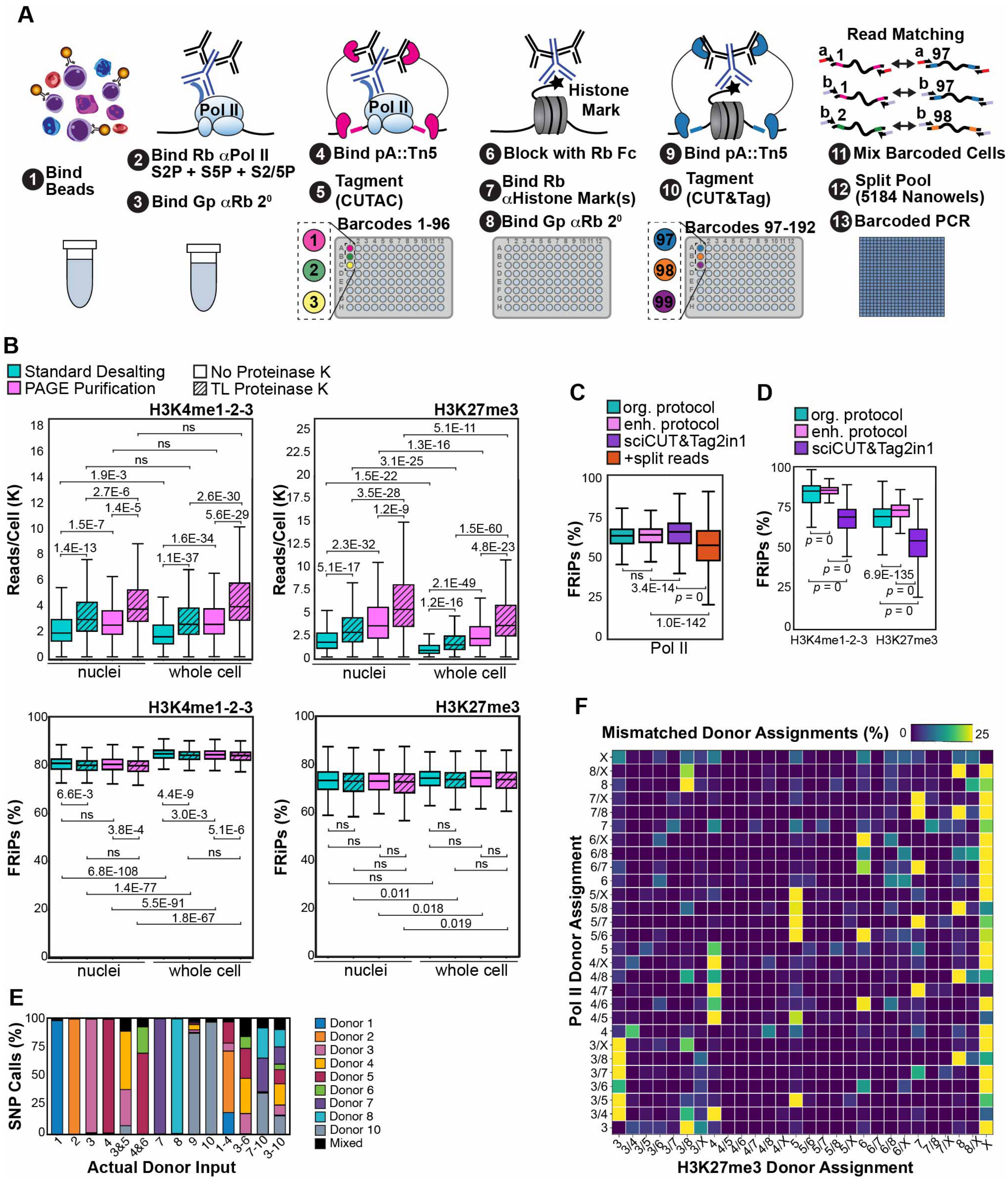

Supplementary Figure 1. An optimized sciCUT&Tag2in1 protocol for profiling human CD34+ HSPCs as whole cells, Related to Figure 1. (A) Schematic overview of the sciCUT&Tag2in1 workflow for joint profiling of RNA Polymerase II and histone modifications in single cells. The method includes two sequential tagmentation steps. The first targeting Pol II

using barcoded pA-Tn5, and the second targeting histone modifications using an orthogonal barcoded pA-Tn5. Cells are pooled and redistributed between steps using split-pool barcoding, allowing simultaneous recovery of both chromatin marks. The upper-right schematic gives three examples of barcodes that match reads from histone marks with Pol II. The outer barcodes (a and b) are introduced by PCR, are nanowell specific and must match. The inner barcodes are introduced during tagmentation and must be from the same well of the 96 well plates used in step 5 and step 10. (B) Boxplots showing reads per cell and FRiPs for H3K4me1-2-3 and H3K27me3 sciCUT&Tag profiles under different protocol optimizations, including standard desalting (blue), PAGE-purified adapters (pink), and with/without thermolabile (TL) Proteinase K treatment (diagonal lines). This optimization increases the number of on-target reads recovered from whole cells. From left to right for H3K4me1-2-3 *n* = 1039, 1084, 916, 935, 2252, 2275, 2070 and 1920 cells. From left to right for H3K27me3 *n* = 1277, 1181, 1312, 1392, 1563, 1562, 1448 and 1508 cells. For boxplots here and throughout: center lines = median, box limits = first and third quartiles, whiskers = 1.5 times the interquartile range (IQR), *p*-values determined by an unpaired students t-test unless otherwise indicated. (C) Boxplots showing the FRiPs for Pol II profiling with the original protocol (*n* = 2,940 cells), enhanced protocol (*n* = 4,439 cells), and sciCUT&Tag2in1 protocol with and without split-barcode reads included (*n* = 70,546 cells). (D) Boxplots showing the FRiPs for H3K4me1-2-3 and H3K27me3 in the original, enhanced and sciCUT&Tag2in1 protocols. For H3K4me1-2-3 *n* = 41,273; 19,329 and 45,335 cells, respectively, and for H3K27me3 *n* = 37,295; 2,900 and 68,476 cells, respectively. (E) Barplot showing SNP-based donor assignment accuracy for samples containing mixtures of 1 to 8 donors at predetermined ratios. Souporcell was unable to distinguish between donors nine and ten. (F) Heatmap showing the distribution of mismatched donor calls across all possible Pol II donor assignments colored by the fraction of mismatches corresponding to each possible H3K27me3 donor assignment. Most mismatches are called as a single donor in one dataset and a doublet that includes that donor in the other dataset.

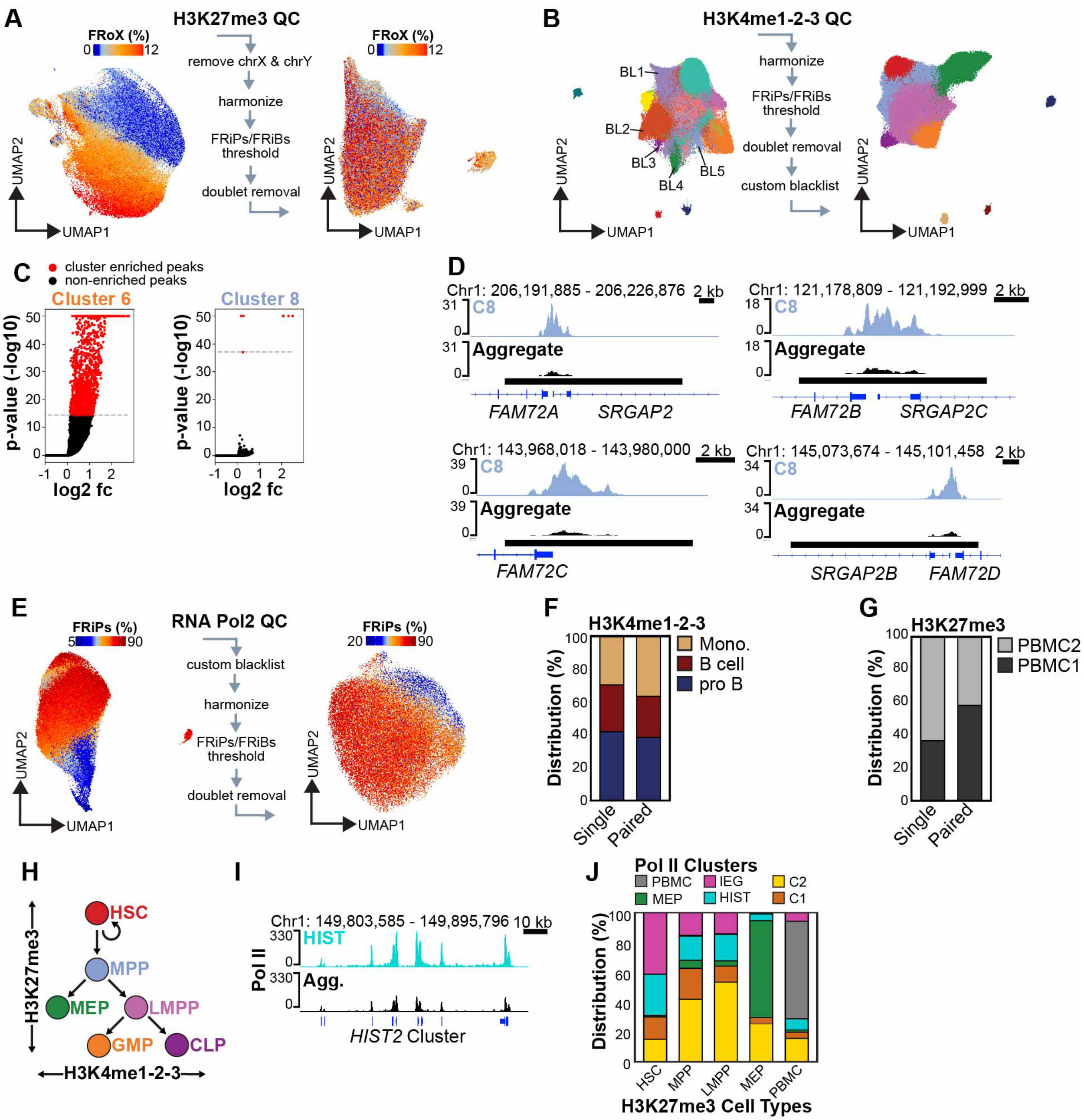

Supplementary Figure 2. Quality control of sciCUT&Tag2in1 single-cell profiles of H3K4me1-2-3, H3K27me3, and RNA Pol II, Related to Figure 2. (A) H3K27me3 UMAP embeddings colored by the Fraction of H3K27me3 reads on chrX. The quality control (QC) workflow for H3K27me3 single-cell profiles included removal of chrX and chrY reads, batch correction, FRiP/Fraction of reads in blacklists (FRiB) thresholding, and removal of clusters enriched for doublets called by Souporcell. (B) H3K4me1-2-3 UMAP embeddings. The left panel shows numerous clusters driven by signal over a limited number of repetitive elements that were subsequently backlisted (BL1-5). The QC workflow for H3K4me1-2-3 single-cell profiles

includes batch correction, FRiP/FRiB thresholding, SNP-based doubled removal and custom blacklisting of repetitive regions that drove artificial clustering. (C) Volcano plots identifying peaks enriched in cluster-specific signal versus non-enriched peaks for representative H3K4me1-2-3 clusters. The small number of H3K4me1-2-3 enriched peaks from Cluster 8 corresponded to the divergent promoters of the *FAM72A-D* and *SRGAP2A-C* gene families, and these peaks were subsequently backlisted. (D) Genome browser plots displaying aggregate H3K4me1-2-3 signal from Cluster 8 over the divergent promoters of the *FAM72A-D* and *SRGAP2A-C* genes. (E) Pol II UMAP embeddings colored by FRiPs. The QC workflow for Pol II single-cell profiles included custom blacklisting, batch correction, FRiP/FRiB thresholding, and SNP-based doublet removal. (F,G) Barplots showing the distribution of major differentiated cell types in single versus paired H3K4me1-2-3 profiles (F), and H3K27me3 profiles (G). (H) Schematic summary showing H3K27me3 informs the location of CD34+ HSCPs along the HSC-differentiation axis while H3K4me1-2-3 distinguish cells at different points in the lineage-commitment axis. (I) Genome browser track showing Pol II enrichment over the *HIST2* cluster. (J) Barplots showing distribution of Pol II clusters across cell types defined by H3K27me3 annotations.

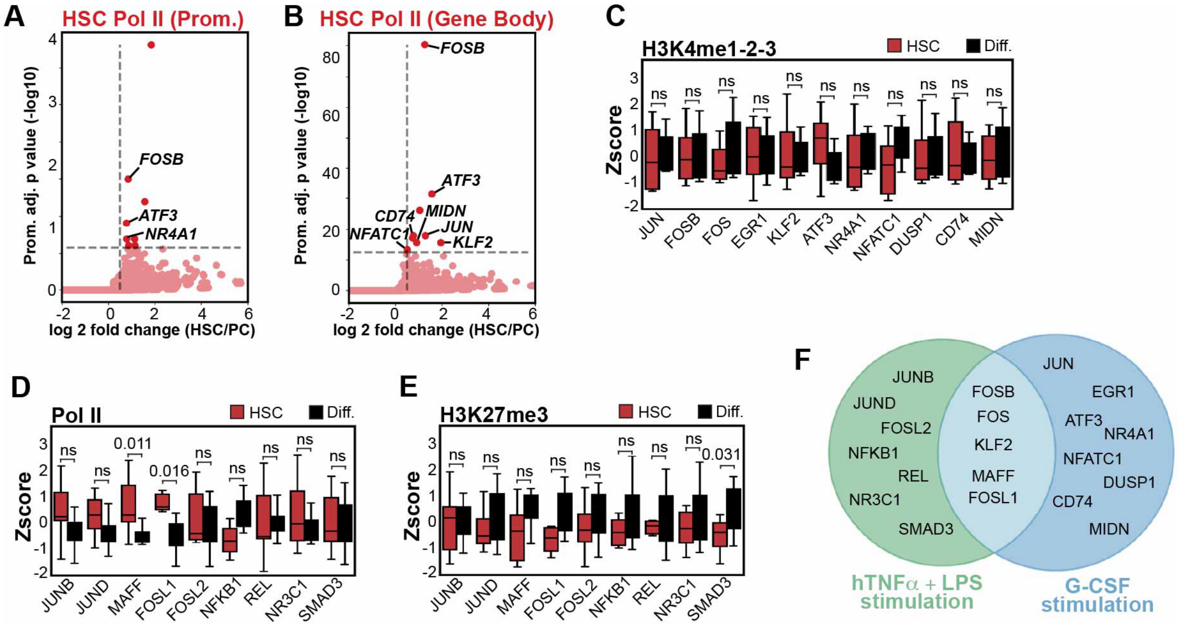

Supplementary Figure 3. G-CSF inflammatory signaling and TNFα induce partially overlapping sets of IEGs in HSCs, Related to Figure 3. (A) Volcano plot showing the enrichment of Pol II over Promoters for all genes in the H3K4me1-2-3 annotated HSC cluster. (B) Same as (A) but showing the enrichment of Pol II over Gene Bodies. (C) Boxplots showing the relative enrichment of H3K4me1-2-3 over the IEGs in HSCs (red) versus the other HSPCs we profiled (Diff., black boxes), *n* = 6 pseudobulk replicates, *p*-values from a one-sided students t-test. (D) Boxplots showing the relative enrichment of Pol II over the top regulatory genes induced by dual inflammatory challenge in Zeng et al. 2023 [1], *n* = 7 pseudobulk replicates paired with H3K4me1-2-3 or H3K27me3, *p*-values from a one-sided students t-test. (E) Same as (D) but showing the relative depletion of H3K27me3 over the inflammatory genes in HSCs (red), *n* = 6 pseudobulk replicates, *p*-values from a one-sided students t-test. (F) Venn Diagram showing the overlap of the top regulatory genes induced during the HSC inflammatory memory response (hTNFα + LPS) and in response to G-CSF stimulation.

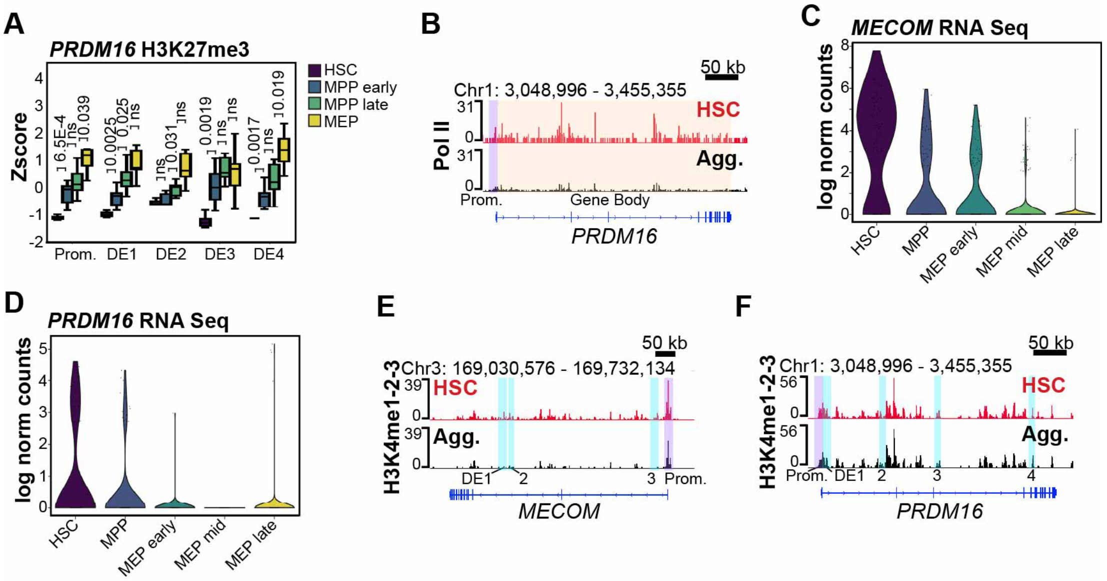

Supplementary Figure 4. Chromatin, Pol II and gene expression dynamics at the self-renewal genes during hematopoietic differentiation, Related to Figure 4. (A) Boxplot showing the relative enrichment of H3K27me3 over the *PRDM16* regulatory elements across the HSC-to-MEP trajectory; *n* = 6 pseudobulk replicates. (B) Genome browser track showing Pol II occupancy across the *PRDM16* locus in HSCs and the aggregate (Agg.) of the other progenitor cell types. (C,D) Violin plots showing the log normalized gene expression counts for *MECOM* (C) and *PRDM16* (D) in cells clustered along the HSC-to-MEP lineage trajectory. (E,F) Genome browser tracks showing H3K4me1-2-3 signal over the *MECOM* locus (E) and *PRDM16* locus (F).

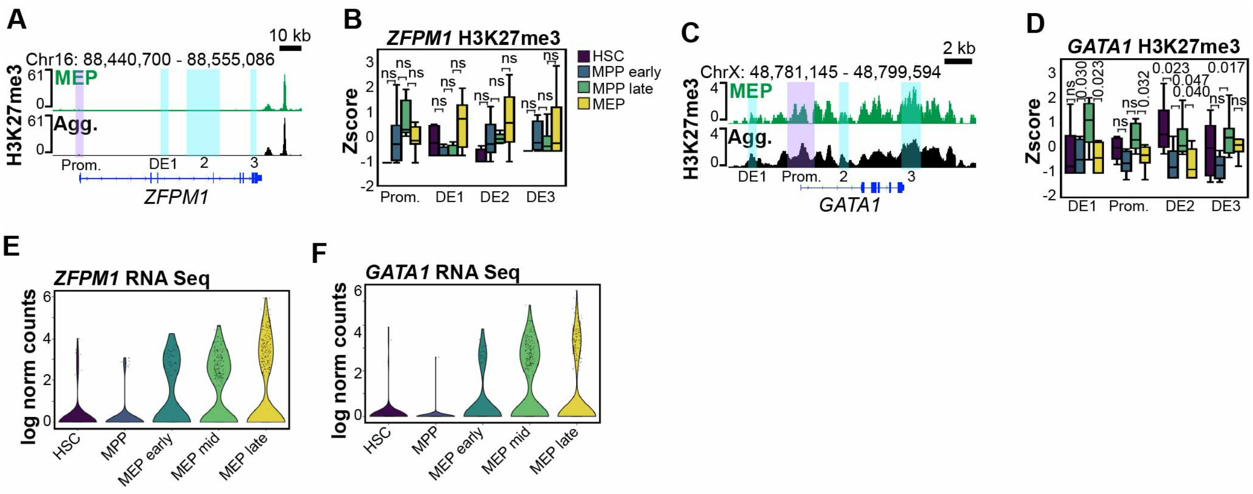

Supplementary Figure 5. H3K27me3 marks the *GATA1* locus but not the *ZFPM1* locus, Related to Figure 5. (A) Genome browser track showing H3K27me3 enrichment across the *ZFPM1* locus in MEPs. (B) Boxplot comparing H3K27me3 z-scores across the *ZFPM1* promoter and distal elements between HSCs and differentiated progenitors; *n* = 6 pseudobulk replicates. (C) Genome browser track showing H3K27me3 signal across the *GATA1* locus in MEPs. (D) Boxplot comparing H3K27me3 z-scores across the *GATA1* promoter and distal elements along the HSC-to-MEP trajectory; *n* = 6 pseudobulk replicates. (E,F) Violin plots showing the log normalized gene expression counts for *ZFPM1* (E) and *GATA1* (F) in cells clustered along the HSC-to-MEP lineage trajectory.

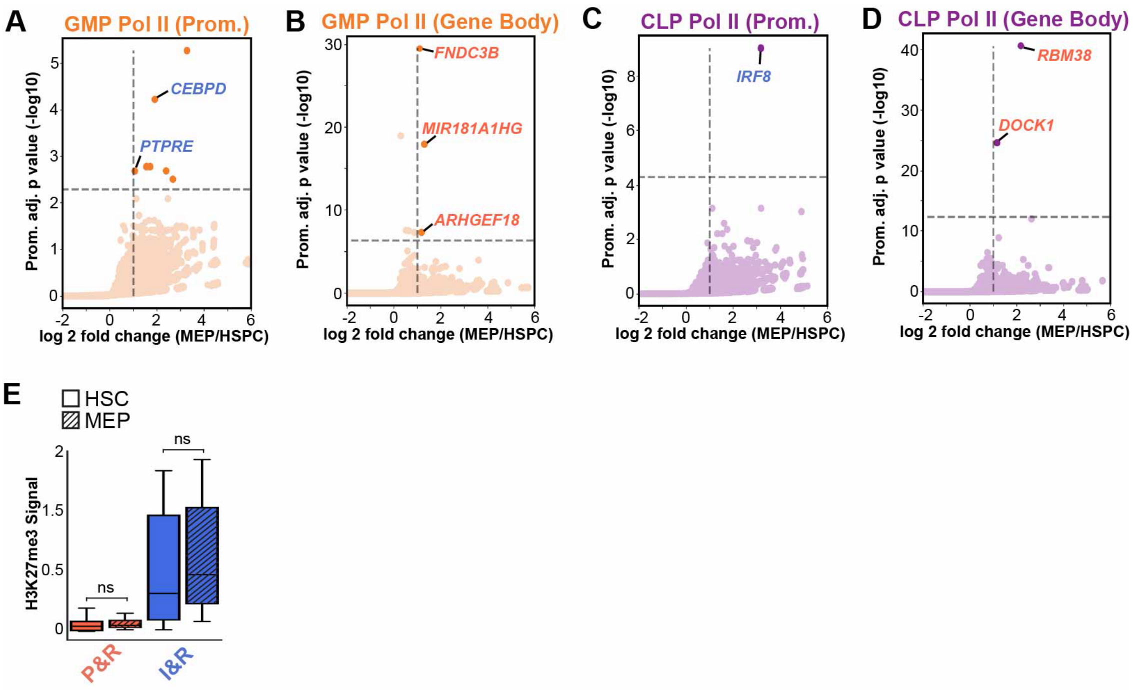

Supplementary Figure 6. Pol II and H3K27me3 dynamics during lineage commitment, Related to Figure 6. (A, B) Volcano plots showing the differential Pol II occupancy in GMPs relative to HSPCs over Promoters (A) and Gene Bodies (B). (C, D) Volcano plots showing the differential Pol II occupancy in CLPs relative to HSPCs over Promoters (C) and Gene Bodies (D). (E) Boxplot showing the H3K27mde3 levels are consistent across pause-and-release (P&R; *n* = 25 genes) and initiate-and-release (I&R; *n* = 19 genes) genes between HSCs and MEPs.

Supplemental References:

1. Zeng, G. X. *et al.* Identification of a human hematopoietic stem cell subset that retains memory of inflammatory stress. *bioRxiv* (2023). https://doi.org/10.1101/2023.09.11.557271
